# Distribution patterns of Chinese Cixiidae (Hemiptera, Fulgoroidea), highlight their high endemic diversity

**DOI:** 10.3897/BDJ.10.e75303

**Published:** 2022-01-24

**Authors:** Yang Luo, Thierry Bourgoin, Jia-Lin Zhang, Ji-Nian Feng

**Affiliations:** 1 Key Laboratory of Plant Protection Resources and Pest Management, Ministry of Education, Entomological Museum, College of Plant Protection, Northwest A&F University, Yangling, Shaanxi 712100, China, Yangling, China Key Laboratory of Plant Protection Resources and Pest Management, Ministry of Education, Entomological Museum, College of Plant Protection, Northwest A&F University, Yangling, Shaanxi 712100, China Yangling China; 2 Institut de Systématique, Évolution, Biodiversité, ISYEB-UMR 7205, MNHN-CNRS-Sorbonne Université-EPHE-Univ. Antilles, Muséum national d’Histoire naturelle, CP 50, 57 rue Cuvier, F-75005, Paris, France Institut de Systématique, Évolution, Biodiversité, ISYEB-UMR 7205, MNHN-CNRS-Sorbonne Université-EPHE-Univ. Antilles, Muséum national d’Histoire naturelle, CP 50, 57 rue Cuvier, F-75005 Paris France

**Keywords:** Checklist, zoogeography region, distribution, species richness, endemism, Cixiidae, China

## Abstract

**Background:**

Cixiidae are small strictly phytophagous hemipteran insects worldwide distributed. Ecology and systematics of Chinese fauna remains poorly investigated. For instance, does their distribution follows the patterns of biogeogaphical distribution established for their host plants or other related-taxa because they are all obligatory phytophagous taxa? Do they follow the usual distributional Chinese realms and boundaries already recognized? Which zoogeographical Chinese regions and connections between them do they depict. To investigate these issues, we provide here a referenced and comprehensive checklist of the 250 cixiid species currently reported from China (77 new records), with their precise distribution at the regional level. In the 8 Chinese main zoogeographical regions usually recognized and 2 adjacent areas, we analyzed further their diversity at the tribal, generic, and specific levels using a non-metric multidimensional scaling and an unweighted pairwise group analysis using an arithmetic mean cluster analyses. The observed distribution patterns shown that an intercalary Sino-Japanese realm is recognisable between the Palaearctic and Oriental realms. At the regional level, the South China region clusters more closely with the Southwest, Central and North China regions. Taiwan, clearly separated from the South China region and mainland China, is more closely related to the Qinghai-Tibet region and Indochina countries. Although Central and South China regions remain close to each other, the Qinghai-Tibet region appears singularly different.

**New information:**

An updated checklist of the 250 Cixiidae species, known to occur in China and counting for 10% of the Chinese planthopper fauna, is presented based on literature, recent collections, and museum records. More than 400 records distributed among the 28 provinces and 8 regions in China are extensively provided, including 77 new records. Of these, more than 80% of the species (205 species, 82%) have been only reported from China, and most of them are endemic species, which could reflects the great diversity degree of the Chinese regions and local biotypes highlights the uniqueness of this fauna. These species are found in 8 Chinese zoogeographical regions: The Taiwan region is the most diversified with 161 species and the highest rate of endemic species (69.57%), followed by South China (78 species, 17.95%), Central China (60 species, 33.33%), Southwest China (43 species, 39.53%), North China (29 species, 34.48%), Qinghai-Tibet region (10 species, 20%), Northeast China (8 species, 12.5%), and 5 species found in the Inner Mongolia-Xinjiang region that are not endemic ones. Endemism was analyzed for each region and repeated for species distribution patterns across them, 9 being bi-regionally and tri-regionally distributed. The South China-Taiwan pattern is the most richest one, followed by the Central-South China-Taiwan pattern. Semonini and Pentastirini tribes are widespread among all the zoological regions, representing respectively 21.20% and 17.20% of all the species, while Cixiini being is the most common tribe with 45.20%, remains absent from the North-Eastern China region. Andini with only 5.20% of the species is distributed in the Sino-Japanese - Oriental Region; Eucarpini (6.40%) and Borysthenini (2.00%) are mainly concentrated in the south of the Qingling Mountain-Huai River. The remaining four tribes, Bennini (0.40%), Briixini (0.80%), Oecleini (1.20%) and Stenophlepsiini (0.40%) are relatively rare and restricted to Taiwan. At the generic level, *Kuvera* (7.2%) is the most widely distributed genus in China while *Cixius*, *Betacixius*, *Kuvera*, *Oecleopsis* and *Andes* are the more diversified. One genus (*Oliparisca)* is distributed only in the Tibet region, while 10 genera are distributed only in the Taiwan region. In addition, nearly half of the genera (16 genera, 48.48%) are distributed south of the Palearctic/Oriental boundary. A non-metric multidimensional scaling and an unweighted pairwise group method analysis using arithmetic mean clustering based on the Jaccard similarity coefficient matrix support a Palaearctic/Sino-Japanese boundary and a South China region closer to the Southwest, Central and North China regions. The Taiwan region appears clearly separated from the South China region and to mainland China, and more closely related to the Qinghai-Tibet region and Indochina countries. The Central and South China regions appear close to each other, but the Qinghai-Tibet region is singularly isolated.

## Introduction

China covers an area of 9,634,057 km^2^, encompassing a area of entire Europe, and spans nearly 50 degrees of latitude from north to south, and more than 60 degrees of longitude from east to west in a world-renowned monsoon region (National Bureau of Statistics of the People’s Republic of China, http://data.stats.gov.cn). Most regions have cold, dry winters and warm, rainy summers, but in combination with the varying topography and terrain conditions, the climate is actually very complex and locally diverse with a wide variety of temperature zones and precipitation gradients (Ren and Wen 2011). Most regions are located in the temperate zone (semi-tropical, warm, mid-range, and cold-temperatures). A small portion of the country is in the tropics and plateau climate zone (the Qinghai-Tibet plateau temperate zone), and northern regions are close to the boreal zone (Jiang 2017). Annual precipitation decreases from the rain-forest of the southeast coast to the Gobi Desert in the northwestern interior (Jiang 2017). An arid humidity zone covers about 31% of the land area (mainly in northwest China). A semi-arid zone covers 22%, a semi-humid zone covers 15%, and the humid zone (32%) is located primarily in the southeast of China (Ge et al. 2013). Geological complexity of China is also significant, particularly with the uplift of the Qinhai-Tibet Plateau, which occurred in the middle of the Eocene era (45-38 Ma) (Zhou et al. 2018). When this complexity is combined with the monsoonal climate evolution, it has created strongly diversified biotopes, isolated by biogeographical barriers that manage dispersal pathways for species, providing new ecological niches, which has driven the recent evolution of plants and animal diversity (Favre et al. 2015, Liu et al. 2017).

**Figure 1.**
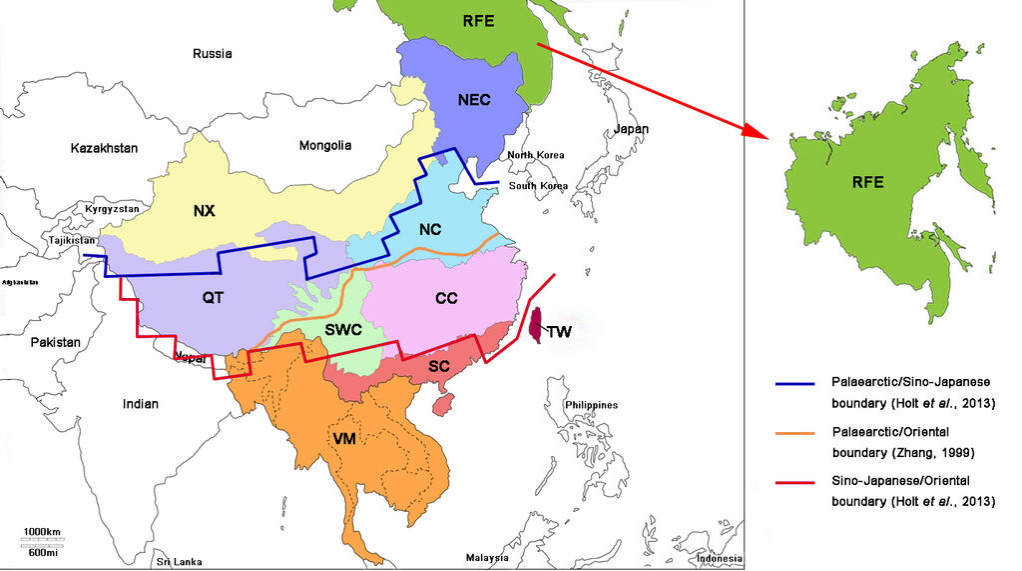
Map of zoogeographical regions of China and adjacent areas. Abbreviations: NEC, Northeast China; NC, North China; NX, Nei Mongol-Xinjiang; QT, Qinghai-Tibet; SWC, Southwest China; CC, Central China; SC, South China; TW, Taiwan; RFE, Russian Far East; VM, Vietnam, Laos, Thailand, Cambodia, Myanmar, Bhutan, Bangladesh and part of Indian.

From a biogeographical point of view, China is usually divided in two parts, the Palearctic realm in the north, and the Oriental one in the south (Sclater 1858, Wallace 1876, Morrone 2015, He et al. 2017). From a zoological perspective, Holt et al. (2013) recently recognized an additional Sino-Japanese realm ranging from west of Tibet to the east of the Japanese archipelago standing between them. Accordingly, three main biogeographical lines cross China (Fig.1): the Palaearctic/Sino-Japanese boundary at about 40–41N, the Palearctic/Oriental line that follows the Qingling Mountain-Huai River, around 32–34N, and the Sino-Japanese/Oriental boundary at 24–25N in Southeastern China.

The family Cixiidae Spinola, 1839 (Hemiptera: Fulgoromorpha), is a numerous and diverse taxon with a world-wide distribution (Holzinger et al. 2002, Bourgoin 2021). It comprises 18.6% of the currently known planthopper species (Bourgoin 2021), and is the largest family of the group. Classical taxonomy has divided the Cixiidae into 3 subfamilies: Borystheninae Emeljanov, 1989, Bothriocerinae Muir, 1923 and Cixiinae Spinola, 1839 (Holzinger et al. 2002). However, recent phylogenetical analyses have shown that these divisions remain artificial and three main lineages should better reflect of evolution of the family: an oecleinian lineage (including Bothriocerini), a cixiinian lineage and a pentastirinian lineage (including Borysthenini) (Luo et al. 2021). Therefore, without including the fossil taxa, Cixiidae are currently divided into 18 tribes, 250 genera, and 2600 species (Bourgoin 2021).

Cixiidae nymphs usually live underground and feed on plant rootlets, whereas the adults feed on the above ground phloem tissues of woody or herbaceous plants and ferns (Wilson 1994, Wheeler 2003), predominaly on Asterales (9.2%), Rosales (7.8%), Fabiales (6.7%), Myrtales (6.5%), Lamiales (5.1%) and Ericales (5.1%) in Eudicots and on Poales (8.3%) in Monocots (Bourgoin 2021). Several cixiidae species are considered to be vectors of plant pathogen including viruses, phytoplasmas and other prokaryotic-like organisms (Wilson 2005).

Although the Cixiidae are one of the larger planthopper families, little is known about their ecology, distribution and host plants. In China, knowledge of this fauna is still fragmented and an overall comprehensive study is lacking. The first contribution was by Melichar (1902) who described 2 genera with 5 species from western China. Matsumura (1914) published 'Die Cixiinen Japans', describing 14 genera and 30 species, mostly from Taiwan. Kato (1932) focused on Northeastern China taxa, and published one new species. The first checklist of Cixiidae from the China mainland was provided by Hu (1935), who listed 11 species in 5 genera, which was updated by Metcalf (1936) in his 'Catalogue of the Homoptera’. Since then, many new species have been added. Jacobi (1944) reported 5 new species from the Fujian province. Fennah (1956) added 6 genera and 17 species from South China. Hori (1982) described 3 new *Betacixius* species from Taiwan. Chou et al. (1985) described 7 species in 4 genera in his "Economic Insect Fauna of China (Fulgoromorpha)". Tsaur provided a series of important contributions to the fauna from Taiwan, describing 155 species in 20 genera (Tsaur and Lee 1987, Tsaur et al. 1988, Tsaur 1989a, Tsaur 1989b, Tsaur 1990a, Tsaur 1990b, Tsaur et al. 1991a, Tsaur et al. 1991b, Tsaur and Hsu 2003, Tsaur 2009). Since then, several papers describing new recent taxonomic discoveries have been published (Wang 1991, Wang 1992, Huang 1995, Hua 2000, Liang 2001, Liang 2005a, Liang 2005b, Guo and Wang 2007, Guo et al. 2009, Guo and Feng 2010, Zhang and Chen 2011a, Zhang and Chen 2011b, Zhang and Chen 2013a, Zhang and Chen 2013b, Ren et al. 2014, Xing and Chen 2014, Bai et al. 2015, Li et al. 2016, Zhi et al. 2017, Zhi et al. 2018a, Zhi et al. 2018b, Luo et al. 2019a, Luo et al. 2019b, Zhi et al. 2019, Zhi et al. 2020a, Zhi et al. 2020b, Zhi et al. 2021).

All of these studies primarily focused on taxonomical treats, with limited ecological and geographical interpretations or evaluations. However, Cixiidae are obligatory phytophagous taxa and therefore directly linked to the distribution of their host plants (Attié et al. 2008). They are generally considered feeding on a variety of plants (Larivière 1999) but more precisley documented, they appears mostly oligiphagous or monphagous (Wilson et al. 1994, Bourgoin 2021). The planthopper and its host-plants are both patterned by the historical biogeography of the areas where they are distributed. How Cixiidae do follow the patterns of biogeogaphical distribution (major biological realms, biogeographical regions) already well established in China? Which boundaries can be identified for Cixiidae and at which taxonomical levels? The aim of this paper is to identify these correlations and to investigate how these zoogeographical regions are connected in China.

This current paper provides the first distribution pattern of the Chinese Cixiidae following current Chinese zoogeographical regions recognized and updated species list of Chinese Cixiidae. Accordingly, the objectives of this paper are: (1) to compare Cixiidae species richness at the level of the Chinese zoogeographical regions and to document their distribution patterns and their endemism in each region, both at the tribal and generic level; (2) to investigate what biogeographical patterns the Cixiidae reflect: are they recognized effectively in a particular Sino-Japanese realm or a simple area of transition between the Palearctic and Oriental realms? (3) to provide a comprehensive species list of the Cixiidae from China.

## Materials and methods

Eight Chinese zoogeographic regions, based on geographic, climatic, and vegetation characteristics (Gao et al. 2017, He et al. 2017), were used for the bio-geographical analyses: Northeast China, North China, Nei Mongol-Xinjiang, Qinghai-Tibet, South China, Central China, Southwest China and the Taiwan region (Fig. 1). Two other regions were added for countries adjacent to China: 1) a south China 'VM region' including Vietnam, Laos, Thailand, Cambodia, Myanmar, Bhutan, Bangladesh, as well as a small portion of India, and 2) a north East China 'Far East region' including a portion of Russia. The map (Fig. 1) was created using the National Earth System Science Data Sharing Infrastructure (http://www.geodata.cn).

**Figure 2.**
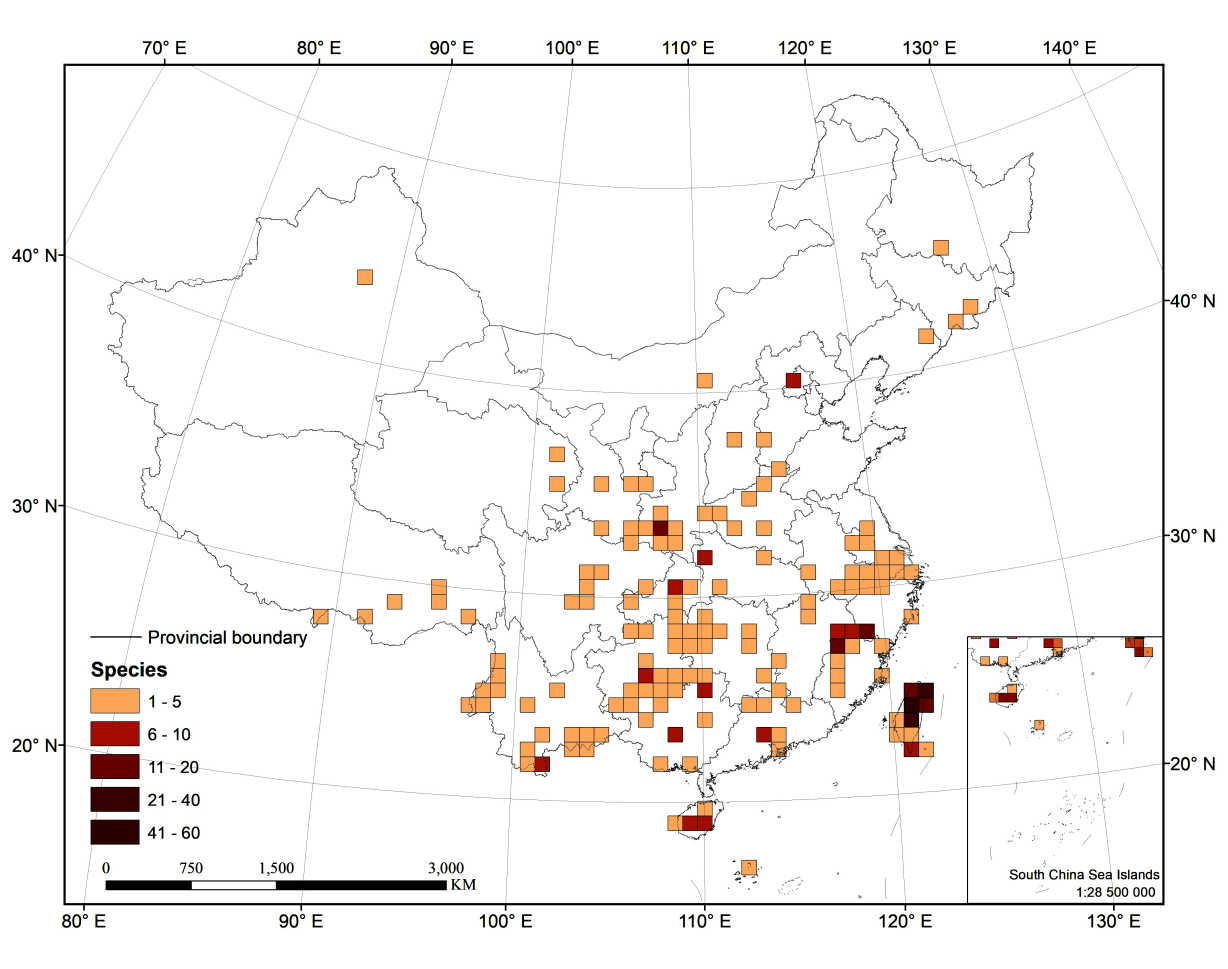
The distribution of species records of Cixiidae species in China.

**Figure 3.**
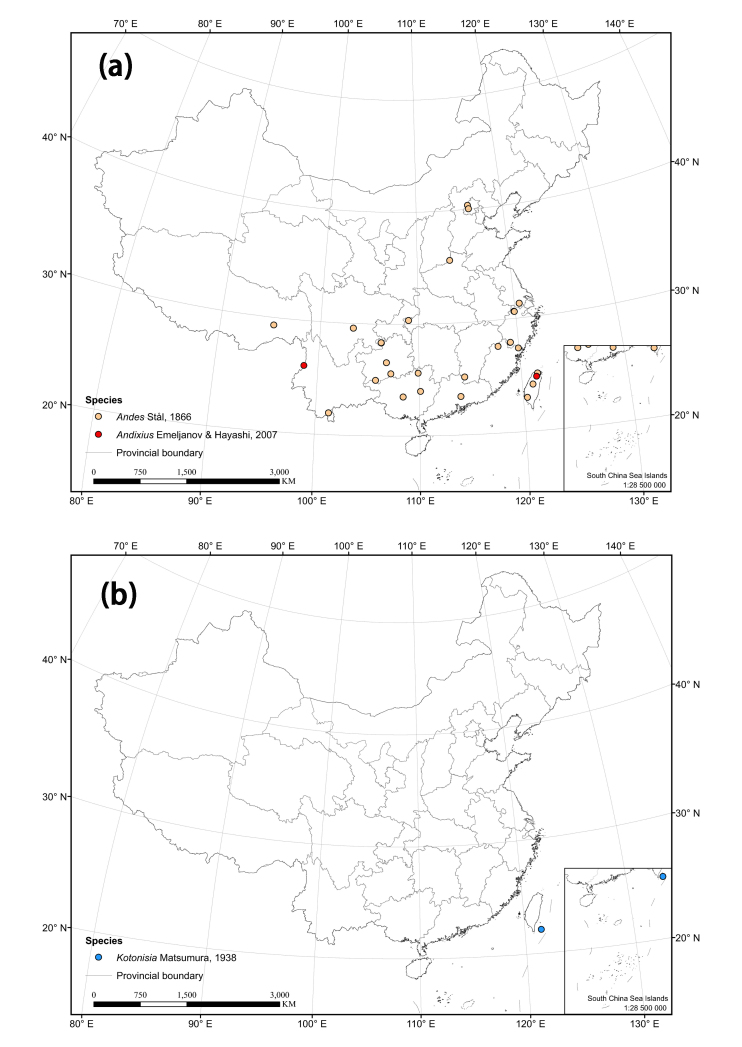
Distribution of records of the tribes Andini and Bennini in China. (a) Andini Emeljanov, 2002; (b) Bennini Metcalf, 1938.

The distribution matrix includes 253 Chinese Cixiidae species (of which 87 species were recorded from museums and the remaining species were recorded from the literature). Among them, 3 species: *Cixiusnarke* Kramer, 1981, *Oliarussplendidulus* Fieber, 1876, and Tachycixius (Tachycixius) pilosus (Olivier, 1791), were excluded from the analyses and checklist because we could not confirm their occurrence in China (no specimens information was found in our inspection of museum specimens in the collections) or because of uncertainties about where they were collected. 48 additional Cixiidae species (Suppl. material 1) from adjacent areas based on literature and FLOW (Bourgoin 2021) were added for the cluster analysis. The observed material information of checklist, as a formatted Excel spreadsheet, are provided here in the supplementary materials: Suppl. material 2. Figure 2 and 3 were generated using ArcGIS Version 10.8 statistical software (URL: https://desktop.arcgis.com/en/system-requirements/latest/arcgis-desktop-system-requirements.htm). The distribution information of the Cixiidae in China was imported into ArcGIS Version 10.8 software, the latitude and longitude of the distribution sites were set as the coordinate attribute elements, and the symbols in the map were set to different colors for distinguishing different genera of the tribes, and finally the maps of the distribution of the tribes and species were exported.

Presence/absence matrices for species and for genera were built for each of the 10 OGUs (physiographical regions as operative geographical units, Crovello 1981). Similarity coefficients use binary data to measure association between OGU. On the basis of a review of similarity coefficients (Shi 1993), the Jaccard's coefficient in NTSYS Version 2.1 software (Rohlf 2000) was used according toLegendre and Legendre (1983) and Rohlf (2000). Clustering of OGUs using the UPGMA algorithm, UPGMA (an unweighted pairwise group method using arithmetic mean) was used to cluster similarities (Legendre and Legendre 1983). Based on the similarity of clustering results, Jaccard's coefficients were analyzed through nonmetric multidimensional scaling (NMDS) according to Kenkel and Orloci (1986).

## Data resources

This publication follows the classical systematic classification based on Holzinger et al. (2002) and Emeljanov (2002) as synthetized and updated in Bourgoin (2021) and Luo et al. (2021). Fossil species are indicated by the symbol (†). The checklist contains information updated up to April, 2021 compiled from scientific papers, book chapters, conference abstracts, theses, and from the FLOW website (Bourgoin 2021). It also includes our own unpublished taxonomic data and original museum specimens information from the following institutions: Shanghai Entomological Museum C.A.S (SEM), Museum of China Agricultural University (CAU), Entomological Museum of Northwest A&F University (NWAFU), Museum of Chinese Academy of Forestry (CAF), Institute of Entomology, Guizhou University (GZU), Chongqing Normal University (CQNU) and Muséum National d'Histoire Naturelle (MNHN). Distribution sets were collected from the original sources with their original latitude and longitude information; Those lacking such information were approximed with the latitude and longitude coordinates of the corresponding administrative center.

## Checklists

### Annotated checklist of Cixiidae from China

#### Cixiidae

Spinola, 1839

B4C00A94-A2A9-5AF0-BC7F-CC11A105A0A1

#### Borystheninae

Emeljanov, 1989

B73E9EF3-B706-5D18-B339-EDE2AEA9C0E4

#### Borysthenes

Stål, 1866

2B46568A-4CD3-5606-9E94-126047FC6C7F

#### Borysthenes acuminatus

Fennah, 1956

028D7029-F346-5B52-AB80-292FB73317B1

Borysthenes acuminatus Fennah, 1956: 459.| Liang, 2005a: 810.

##### Distribution

China: Hubei (Liang 2005a).

#### Borysthenes deflexus

Fennah, 1956

C2E9C508-7520-5142-8D8B-B39AA58BC18D

Borysthenes deflexus Fennah, 1956: 460.| Liang, 2005a: 810.

##### Distribution

China: Guangdong (Fennah 1956).

#### Borysthenes emarginatus

Fennah, 1956

F386DF7E-C858-5CF3-A653-1D02AEE8B4CC

Borysthenes emarginatus Fennah, 1956: 461.| Liang, 2005a: 810.

##### Distribution

China: Guangdong (Fennah 1956).

#### Borysthenes lacteus

Tsaur & Lee, 1987

A67A43AE-A1D8-558E-BD81-93957EC5E849

Borysthenes lacteus Tsaur & Lee, 1987: 9.

##### Distribution

China: Taiwan (Tsaur and Lee 1987).

#### Borysthenes maculatus

(Matsumura, 1914)

6977B388-77CF-513F-82E8-94DBD773804A

Barma maculata Matsumura, 1914: 430.| *Bolysthenes* (sic) *guttatus* Kato, 1933: 468.| *Borysthenesmaculatus* (Matsumura, 1914), Fennah, 1956: 459.| Chou, 1985: 26.| Tsaur & Lee, 1987: 8.| Liang, 2005a: 810| Liang, 2005b: 429.| Hayashi & Fujinuma, 2016: 326.

##### Distribution

China: Fujian (Liang 2005a, Liang 2005b), Hainan, Hunan, Guangxi, Sichuan, Taiwan (Tsaur and Lee 1987); Japan: Nansei-shoto (Hayashi and Fujinuma 2016).

##### Notes

New record: China: Hainan (Diaoluo Mountain).

#### Cixiinae

Spinola, 1839

9E5B213C-EC21-53E2-B747-2B9266786882

#### Andini

Emeljanov, 2002

C7C24FC8-F355-5C87-8845-4F4C57AAECC5

#### Andes

Stål, 1866

89F4DF6B-2313-567F-B6C9-A37B060AB93A

#### Andes formosanus

(Matsumura, 1914)

9AC85C00-E651-5459-AA38-525D6DADFA62

Brixia formosana Matsumura, 1914: 432.| *Andesformosanus* (Matsumura, 1914), in Tsaur et al., 1991a: 70.

##### Distribution

China: Fujian, Sichuan, Taiwan (Matsumura 1914).

##### Notes

New record: China: Fujian (Wuyi Mountain).

#### Andes hemina

Fennah, 1978

0024AB39-4781-5128-80C0-FEA1B5CD49F2

Andes hemina Fennah, 1978: 209.

##### Distribution

China: Yunnan; Malaysia: Kuala Lumpur, Kedah (Fennah 1978); Vietnam: Ninh Bình (Fennah 1978).

##### Notes

New record: China: Yunnan (Menglun).

#### Andes lachesis

Fennah, 1956

66EEBBE7-98FD-5990-867C-7D49129C5B4E

Andes lachesis Fennah, 1956: 447.

##### Distribution

China: Zhejiang (Fennah 1956).

#### Andes luzonensis

Tsaur & Hsu, 1991

C58CAA76-0D41-5664-84DA-0DEBBD4FB6CC

Andes luzonensis Tsaur & Hsu in Tsaur et al., 1991a: 72.

##### Distribution

China: Zhejiang, Taiwan (Tsaur et al. 1991a).

#### Andes marmoratus

(Uhler, 1896)

9B684D7D-4B4F-54B7-8B9A-F867FE65AD74

Metabrixia marmorata Uhler, 1896: 280.| *Brixiamarmorata* (Uhler, 1896), Matsumura, 1914: 431.| *Andesmarmorata* (Uhler, 1896), Chou, 1985: 24.| Liang, 2005b: 429.| Hayashi & Fujinuma, 2016: 323.

##### Distribution

China: Beijing (Liang 2005b), Henan, Jiangsu, Zhejiang, Guangxi, Guizhuo; Japan: Hokkaido, Honshu, Shikoku, Kyushu, Tsushima Island (Palaearctic) (Hayashi and Fujinuma 2016); Russia: Far East.

##### Notes

New record: China: Jiangsu (Suzhou), Zhejiang (Taishun).

#### Andes noctua

Fennah, 1956

0E353325-7094-52D9-B5B5-C539238C781E

Andes noctua Fennah, 1956: 446.| Zhang, 2008: 33.

##### Distribution

China: Beijing, Henan, Hubei (Fennah 1956), Guizhou.

##### Notes

New record: China: Beijing (Mentougou), Henan (Huixian), Hubei (Lichuan).

#### Andes notatus

Tsaur & Hsu, 1991

E33759D0-0393-507D-90B5-50ADC287AB45

Andes notatus Tsaur & Hsu in Tsaur et al., 1991a: 70.

##### Distribution

China: Guangxi, Tibet, Taiwan (Tsaur et al. 1991a).

##### Notes

New record: China: Guangxi (Jinxiu, Longsheng), Tibet (Motuo).

#### Andes othrepte

Fennah, 1956

720922F3-6F07-5EE5-9B81-3EBEB320F675

Andes othrepte Fennah, 1956: 445.

##### Distribution

China: Hong Kong (Fennah 1956).

#### Andes truncatus

Fennah, 1978

7698DE80-07B3-51BF-9357-9DA2DA770498

Andes truncatus Fennah, 1978: 208.

##### Distribution

China: Guizhou, Zhejiang; Vietnam: Ninh Bình (Fennah 1978).

##### Notes

New record: China: Zhejiang (Fengyang Mountain).

#### Andes unicinatus

Fennach, 1956

57BB74AA-F3FC-5EF0-A376-857C228F2DBE

Andes unicinatus Fennach, 1956: 444.| Zhang, 2008: 38.

##### Distribution

China: Guangdong, Sichuan (Fennah 1956).

#### Andixius

Emeljanov & Hayashi, 2007

120AAE2E-AC65-59B3-A453-5B17DE075BA1

#### Andixius longispinus

Zhi & Chen, 2018

B0494B80-72AB-567F-9236-B5B172198D7E

Andixius longispinus Zhi & Chen in Zhi et al., 2018b: 57.

##### Distribution

China: Yunnan (Zhi et al. 2018b).

#### Andixius trifurcus

Zhi & Chen, 2018

A4A12AE6-C39B-5BE3-8CB3-3AF6743D8F32

Andixius trifurcus Zhi & Chen, in Zhi et al., 2018b: 60.

##### Distribution

China: Yunnan (Zhi et al. 2018b).

#### Andixius venustus

Tsaur & Hsu, 1991

E68BD9D9-FAAE-5C34-ADCE-D72030976C66

Brixia venusta Tsaur & Hsu in Tsaur et al., 1991a: 65.| *Andixiusvenustus* (Tsaur & Hsu, 1991), Emeljanov & Hayashi, 2007: 129.

##### Distribution

China: Taiwan (Tsaur et al. 1991a).

#### Bennini

Metcalf, 1938

84BFA855-3303-5C5F-9206-31E41732A775

#### Kotonisia

Matsumura, 1938

ABCCB77F-E995-5B39-9313-3249C2E63B72

#### Kotonisia kanoi

(Matsumura, 1938)

DA5A57E8-56C2-5192-A719-885E735D66B2

Benna kanoi Matsumura, 1938: 152.| *Bennaformosana* (Nast), Tsaur, 1988: 76.| *Kotonisiakanoi* (Matsumura, 1938), Tsaur, 2009: 67.| *Kotonisiakanoi* (Matsumura, 1938), Hoch, 2013: 174.

##### Distribution

China: Taiwan (Tsaur 2009).

#### Brixiini

Emeljanov, 2002

336F0E07-09EF-565E-BA6C-E0FEB7F87020

#### Brixia

Stål, 1859

50004930-32B2-510F-9B2F-83BDFBBA2311

#### Brixia ocellata

Matsumura, 1914

793EB166-9F6A-569B-9D64-952431BC1F74

Brixia ocellata Matsumura, 1914: 433.

##### Distribution

China: Taiwan (Matsumura 1914).

#### Brixia neglecta

Van Stalle, 1983

3BFA30B4-B44D-55FC-880E-155DA38871FE

Brixia neglecta Van Stalle, 1983: 272.

##### Distribution

China: Taiwan.

#### Cixiini

Spinola, 1839

E6EA5E4F-334B-552A-94DF-DF61D4A51544

#### Ankistrus

Tsaur & Hsu, 1991

0A7B2063-4DEA-50A9-90B1-3B8725938217

#### Ankistrus basalis

Tsaur & Hsu, 1991

B3FFF07B-EEFA-582A-93AA-5A80B4FD2439

Ankistrus basalis Tsaur & Hsu in Tsaur et al., 1991a: 19.

##### Distribution

China: Taiwan (Tsaur et al. 1991a).

#### Ankistrus choui

Tsaur & Hsu, 1991

A8401FC3-6D09-5220-97F0-F48084260A53

Ankistrus choui Tsaur & Hsu in Tsaur et al., 1991a: 12.

##### Distribution

China: Taiwan (Tsaur et al. 1991a).

#### Ankistrus guttatus

Tsaur & Hsu, 1991

CFAE1F43-0443-5A7F-839F-84A5191AA2A3

Ankistrus guttatus Tsaur & Hsu in Tsaur et al., 1991a: 17.

##### Distribution

China: Taiwan (Tsaur et al. 1991a).

#### Ankistrus montanus

Tsaur & Hsu, 1991

ADC7EF88-3DC9-5078-B205-FE5BD236B4CB

Ankistrus montanus Tsaur & Hsu in Tsaur et al., 1991a: 9.

##### Distribution

China: Taiwan (Tsaur et al. 1991a).

#### Ankistrus pini

Tsaur & Hsu, 1991

42C5C12A-F647-54B2-96B0-02A17D2A0B52

Ankistrus pini Tsaur & Hsu in Tsaur et al., 1991a: 9.

##### Distribution

China: Taiwan (Tsaur et al. 1991a).

#### Ankistrus taoi

Tsaur & Hsu, 1991

00CE480F-E585-5CA7-9B54-D5231F94ADA7

Ankistrus taoi Tsaur & Hsu in Tsaur et al., 1991a: 14.

##### Distribution

China: Taiwan (Tsaur et al. 1991a).

#### Ankistrus varius

Tsaur & Hsu, 1991

1833FAE1-0666-5EC1-920E-D49754D6C0BA

Ankistrus varius Tsaur & Hsu in Tsaur et al., 1991a: 14.

##### Distribution

China: Taiwan (Tsaur et al. 1991a).

#### Cixius

Latreille, 1804

63F8E04D-E8EC-5994-A9FF-C96B872A4F1E

#### Cixius aculeatus

Tsaur & Hsu, 1991

F82D2B1F-56DD-519C-B332-5B1DC87CA661

Cixius aculeatus Tsaur & Hsu in Tsaur et al., 1991b: 199.

##### Distribution

China: Taiwan (Tsaur et al. 1991b).

#### Cixius acutus

Tsaur & Hsu, 1991

1DE9C415-83D2-524E-95E8-3E49815585BB

Cixius acutus Tsaur & Hsu in Tsaur et al., 1991b: 204.

##### Distribution

China: Taiwan (Tsaur et al. 1991b).

#### Cixius aduncus

Tsaur & Hsu, 1991

A53BE908-C0CE-5E10-BEEB-A4C36C71A9B1

Cixius aduncus Tsaur & Hsu in Tsaur et al., 1991b: 239.

##### Distribution

China: Taiwan (Tsaur et al. 1991b).

#### Cixius alpinus

Tsaur & Hsu, 1991

9C8C06C6-E0A6-538A-A11B-6131AEF6E750

Cixius alpinus Tsaur & Hsu in Tsaur et al., 1991b: 242.

##### Distribution

China: Taiwan (Tsaur et al. 1991b).

#### Cixius anmashanus

Tsaur & Hsu, 1991

54DAD350-AFBA-5AA4-99B6-A1EDA4853CAA

Cixius anmashanus Tsaur & Hsu in Tsaur et al., 1991b: 266.

##### Distribution

China: Taiwan (Tsaur et al. 1991b).

#### Cixius aquilonius

Tsaur & Hsu, 1991

C027BBDF-3194-54D5-BE52-550EA2518DEB

Cixius aquilonius Tsaur & Hsu in Tsaur et al., 1991b: 260.

##### Distribution

China: Taiwan (Tsaur et al. 1991b).

#### Cixius arisanus

Tsaur & Hsu, 1991

A21D1D76-2E7D-53F4-B962-10172C19830C

Cixius arisanus Matsumura, 1914: 386.| Tsaur et al., 1991b: 185.

##### Distribution

China: Zhejiang, Hainan, Taiwan (Tsaur et al. 1991b).

##### Notes

New record: China: Zhejiang (Fengyang Mountain).

#### Cixius bicolor

Matsumura, 1914

A4F985B9-6F8A-5828-8D27-32E73453CC6C

Cixius bicolor Matsumura, 1914: 395.| Esaki, 1932: 1773.| Tsaur et al., 1991b: 175.

##### Distribution

China: Taiwan (Tsaur et al. 1991b), Hainan; Japan.

##### Notes

New record: China: Hainan (Limuling).

#### Cixius bidentis

Tsaur & Hsu, 1991

71CC12A3-459B-53ED-8B1A-610D0475115B

Cixius bidentis Tsaur & Hsu in Tsaur et al., 1991b: 279.

##### Distribution

China: Taiwan (Tsaur et al. 1991b).

#### Cixius brochus

Tsaur & Hsu, 1991

B7739556-840C-5FA6-A290-758760E95830

Cixius brochus Tsaur & Hsu in Tsaur et al., 1991b: 222.

##### Distribution

China: Taiwan (Tsaur et al. 1991b).

#### Cixius broncus

Tsaur & Hsu, 1991

9E28851C-50AC-56D8-8C56-23CF6C1CC6F5

Cixius broncus Tsaur & Hsu in Tsaur et al., 1991b: 233.

##### Distribution

China: Taiwan (Tsaur et al. 1991b).

#### Cixius capillatus

Tsaur & Hsu, 1991

F70EA667-CCDC-5CC3-A682-BDA49F7C69C6

Cixius capillatus Tsaur & Hsu in Tsaur et al., 1991b: 208.

##### Distribution

China: Taiwan (Tsaur et al. 1991b).

#### Cixius cathetus

Tsaur & Hsu, 1991

9C1AB30C-B3A9-5913-863C-B10EC5ABE256

Cixius cathetus Tsaur & Hsu in Tsaur et al., 1991b: 275.

##### Distribution

China: Taiwan (Tsaur et al. 1991b).

#### Cixius chituanus

Tsaur & Hsu, 1991

6D37E211-4B8E-536B-B94B-64E94AD3305C

Cixius chituanus Tsaur & Hsu in Tsaur et al., 1991b: 236.

##### Distribution

China: Taiwan (Tsaur et al. 1991b).

#### Cixius chouorum

Tsaur & Hsu, 1991

7BF767BE-3089-530A-B914-D6983DD76AF3

Cixius chouorum Tsaur & Hsu in Tsaur et al., 1991b: 225.

##### Distribution

China: Taiwan (Tsaur et al. 1991b).

#### Cixius chydaeus

Tsaur & Hsu, 1991

62F98740-FD80-5391-ADBB-1D5D59F9A387

Cixius chydaeus Tsaur & Hsu in Tsaur et al., 1991b: 252.

##### Distribution

China: Taiwan (Tsaur et al. 1991b).

#### Cixius circinatus

Tsaur & Hsu, 1991

6B64193C-00A6-56C6-8A08-CD544558AA94

Cixius circinatus Tsaur & Hsu in Tsaur et al., 1991b: 202.

##### Distribution

China: Taiwan (Tsaur et al. 1991b).

#### Cixius circulus

Tsaur & Hsu, 1991

2E89B9FF-45E6-5F5D-A4CE-0A311C7496B8

Cixius circulus Tsaur & Hsu in Tsaur et al., 1991b: 219.

##### Distribution

China: Taiwan (Tsaur et al. 1991b).

#### Cixius communis

Tsaur & Hsu, 1991

C565BCEA-ED14-5535-9312-8FD157E683DC

Cixius communis Tsaur & Hsu in Tsaur et al., 1991b: 200.

##### Distribution

China: Guangxi, Zhejiang, Taiwan (Tsaur et al. 1991b).

##### Notes

New record: China: Guangxi (Nanning), Zhejiang (Fengyang Mountain).

#### Cixius curvus

Tsaur & Hsu, 1991

4D57F482-9BA4-573E-B697-337992640B15

Cixius curvus Tsaur & Hsu in Tsaur et al., 1991b: 183.

##### Distribution

China: Taiwan (Tsaur et al. 1991b).

#### Cixius cyclus

Tsaur & Hsu, 1991

38EF48CC-54A4-58EA-9027-4A2422E83CEB

Cixius cyclus Tsaur & Hsu in Tsaur et al., 1991b: 256.

##### Distribution

China: Taiwan (Tsaur et al. 1991b).

#### Cixius deflexus

Tsaur & Hsu, 1991

89078885-E950-5920-8BAA-36A7999F3789

Cixius deflexus Tsaur & Hsu in Tsaur et al., 1991b: 300.

##### Distribution

China: Taiwan (Tsaur et al. 1991b).

#### Cixius denotatus

Tsaur & Hsu, 1991

2169A1BA-70B3-5912-A6F9-0C24B3B3FCC5

Cixius denotatus Tsaur & Hsu in Tsaur et al., 1991b: 294.

##### Distribution

China: Taiwan (Tsaur et al. 1991b).

#### Cixius dentatus

Tsaur & Hsu, 1991

89BE279B-7B77-528E-90F3-C95F89EBF639

Cixius dentatus Tsaur & Hsu in Tsaur et al., 1991b: 254.

##### Distribution

China: Taiwan (Tsaur et al. 1991b).

#### Cixius denticulatus

Tsaur & Hsu, 1991

795BAA48-4D68-5314-BDC6-E4BCBC212E0E

Cixius denticulatus Tsaur & Hsu in Tsaur et al., 1991b: 180.

##### Distribution

China: Taiwan (Tsaur et al. 1991b).

#### Cixius diductus

Tsaur & Hsu, 1991

41AF7679-1851-5749-87F5-7D6F4E29147C

Cixius diductus Tsaur & Hsu in Tsaur et al., 1991b: 241.

##### Distribution

China: Taiwan (Tsaur et al. 1991b).

#### Cixius dilatus

Tsaur & Hsu, 1991

1BF55B8B-42CC-5214-86AD-7B16086BAC3C

Cixius dilatus Tsaur & Hsu in Tsaur et al., 1991b: 286.

##### Distribution

China: Taiwan (Tsaur et al. 1991b).

#### Cixius discretus

Li, Liu, Ren, Li & Yao, 2016

2B9B60C4-66FE-53FB-9EFD-C5D490D54301

Cixius discretus † Li, Liu, Ren, Li & Yao in Li et al., 2016: 2.

##### Distribution

China: Qinghai (Li et al. 2016).

##### Notes

Fossil species

#### Cixius elegantulus

Tsaur & Hsu, 1991

8B386C62-4D09-548A-B867-DC7DE93FEA81

Cixius elegantulus Tsaur & Hsu in Tsaur et al., 1991b: 244.

##### Distribution

China: Taiwan (Tsaur et al. 1991b).

#### Cixius elongatus

Tsaur & Hsu, 1991

832A46BC-9E39-5D42-B76D-E904FD2F1306

Cixius elongatus Tsaur & Hsu in Tsaur et al., 1991b: 206.

##### Distribution

China: Taiwan (Tsaur et al. 1991b).

#### Cixius fangi

Tsaur & Hsu, 1991

28AC5137-D465-5958-986B-DE437A348BE2

Cixius fangi Tsaur & Hsu in Tsaur et al., 1991b: 180.

##### Distribution

China: Taiwan (Tsaur et al. 1991b).

#### Cixius flavescens

Matsumura, 1914

4FDBA5A1-8679-5125-A540-29A8B9BDD162

Cixius flavescens Matsumura, 1914: 405.

##### Distribution

China: Shaanxi, Taiwan (Matsumura 1914).

##### Notes

New record: China: Shaanxi (Hanzhong).

#### Cixius furvus

Tsaur & Hsu, 1991

CE2F1785-CAB0-5626-BC48-12A9307A83E3

Cixius furvus Tsaur & Hsu in Tsaur et al., 1991b: 285.

##### Distribution

China: Taiwan (Tsaur et al. 1991b).

#### Cixius fustis

Tsaur & Hsu, 1991

617AC248-F0A1-5211-BC61-7778D7000949

Cixius fustis Tsaur & Hsu in Tsaur et al., 1991b: 228.

##### Distribution

China: Taiwan (Tsaur et al. 1991b).

#### Cixius galeolus

Fennah, 1956

937C701D-3887-5CE9-9B7A-A6492E4B5F25

Cixius galeolus Fennah, 1956: 449.

##### Distribution

China: Guangdong (Fennah 1956).

#### Cixius gladius

Tsaur & Hsu, 1991

1D9DFCBC-67AE-5F49-B6C1-9E7AAC20CBD1

Cixius gladius Tsaur & Hsu in Tsaur et al., 1991b: 225

##### Distribution

China: Taiwan (Tsaur et al. 1991b).

#### Cixius habunus

Tsaur & Hsu, 1991

693B3781-428F-5BBF-BEF5-A26EBBBCB8B2

Cixius habunus Tsaur & Hsu in Tsaur et al., 1991b: 188.

##### Distribution

China: Taiwan (Tsaur et al. 1991b).

#### Cixius hopponis

Matsumura, 1914

906EA96A-5DB2-5A3E-BC3A-2D7C4BEA9FE7

Cixius hopponis Matsumura, 1914: 399| Tsaur et al., 1991b: 195.

##### Distribution

China: Taiwan (Tsaur et al. 1991b).

#### Cixius hsui

Tsaur & Hsu, 1991

1C8DCE35-111D-502C-96D3-B510EB59FC6D

Cixius hsui Tsaur & Hsu in Tsaur et al., 1991b: 256.

##### Distribution

China: Taiwan (Tsaur et al. 1991b).

#### Cixius hueisunus

Tsaur & Hsu, 1991

C19B0B61-9836-5802-B8E7-1CF2059FC936

Cixius hueisunus Tsaur & Hsu in Tsaur et al., 1991b: 279.

##### Distribution

China: Taiwan (Tsaur et al. 1991b).

#### Cixius inaffectus

Tsaur & Hsu, 1991

6EC1BDE8-C72C-5581-AE3A-E244D73E3612

Cixius inaffectus Tsaur & Hsu in Tsaur et al., 1991b: 212.

##### Distribution

China: Taiwan (Tsaur et al. 1991b).

#### Cixius incisus

Tsaur & Hsu, 1991

E8542DF3-D523-5DFB-BA1D-38DF7AF8DFF4

Cixius incisus Tsaur & Hsu in Tsaur et al., 1991b: 219.

##### Distribution

China: Taiwan (Tsaur et al. 1991b).

#### Cixius inflatus

Tsaur & Hsu, 1991

EEDB50D3-ACCA-574F-B8D3-86FC1DDBB003

Cixius inflatus Tsaur & Hsu in Tsaur et al., 1991b: 272.

##### Distribution

China: Taiwan (Tsaur et al. 1991b).

#### Cixius kommonis

Matsumura, 1914

9B56CBD4-47B2-5779-AD02-9EB9DE5DBDD7

Cixius kommonis Matsumura, 1914: 401| Tsaur et al., 1991b: 301.

##### Distribution

China: Taiwan (Tsaur et al. 1991b).

#### Cixius kukuanus

Tsaur & Hsu, 1991

C431752E-E30C-5787-A439-0AE84723D8C7

Cixius kukuanus Tsaur & Hsu in Tsaur et al., 1991b: 269.

##### Distribution

China: Taiwan (Tsaur et al. 1991b).

#### Cixius kuyanyanus

Matsumura, 1914

46245028-261A-5C17-A1A3-65C394A16CD9

Cixius kuyanyanus Matsumura, 1914: 398.

##### Distribution

China: Taiwan (Tsaur et al. 1991b).

#### Cixius laboriosus

Tsaur & Hsu, 1991

A6C62A6B-DFD0-513F-A06A-FD30895AE82A

Cixius laboriosus Tsaur & Hsu in Tsaur et al., 1991b: 272.

##### Distribution

China: Taiwan (Tsaur et al. 1991b).

#### Cixius latus

Tsaur & Hsu, 1991

94FDE82A-B965-5F43-9CB4-F4DD0F64906A

Cixius latus Tsaur & Hsu in Tsaur et al., 1991b: 248.

##### Distribution

China: Taiwan (Tsaur et al. 1991b).

#### Cixius leei

Tsaur & Hsu, 1991

ABC7D7D4-15B2-58E0-A3F4-5D975E2C284F

Cixius leei Tsaur & Hsu in Tsaur et al., 1991b: 282.

##### Distribution

China: Zhejiang, Taiwan (Tsaur et al. 1991b).

##### Notes

New record: China: Zhejiang (Feng Mountain).

#### Cixius linorum

Tsaur & Hsu, 1991

E8C80E02-88BD-5B5D-911E-7B17961A376B

Cixius linorum Tsaur & Hsu in Tsaur et al., 1991b: 216.

##### Distribution

China: Taiwan (Tsaur et al. 1991b).

#### Cixius luridus

Tsaur & Hsu, 1991

D7079E9D-E8C4-53D8-97C3-9D8410272F6E

Cixius luridus Tsaur & Hsu in Tsaur et al., 1991b: 264.

##### Distribution

China: Taiwan (Tsaur et al. 1991b).

#### Cixius maculosus

Tsaur & Hsu, 1991

46E7B3B3-2910-57D1-B3E6-73804DC7C430

Cixius maculosus Tsaur & Hsu in Tsaur et al., 1991b: 188.

##### Distribution

China: Taiwan (Tsaur et al. 1991b).

#### Cixius meifengensis

Tsaur & Hsu, 1991

65875310-07BB-526C-ACAE-FE590576611D

Cixius meifengensis Tsaur & Hsu in Tsaur et al., 1991b: 208.

##### Distribution

China: Taiwan (Tsaur et al. 1991b).

#### Cixius montosus

Tsaur & Hsu, 1991

735383BE-2B54-5328-8206-0BA34A9C2D01

Cixius montosus Tsaur & Hsu in Tsaur et al., 1991b: 205.

##### Distribution

China: Taiwan (Tsaur et al. 1991b).

#### Cixius mukwanus

Tsaur & Hsu, 1991

C666BBEA-8413-532F-AFF4-6B7A326171EF

Cixius mukwanus Tsaur & Hsu in Tsaur et al., 1991b: 176.

##### Distribution

China: Fujian, Taiwan (Tsaur et al. 1991b).

##### Notes

First record: China: Fujian (Wuyi Mountain).

#### Cixius nervosus

(Linné, 1758)

9AE644EE-4940-5448-A85D-4D0230EFD460

Cicada nervosa Linné, 1758: 437.| *Cixiusnervosus* (Linné, 1758), Beirne, 1951: 315.| Kramer, 1981: 8.| Bartlett et al., 2014: 90.| Hayashi & Fujinuma, 2016: 324.

##### Distribution

China: Ningxia; Algeria (Nast 1972, Holzinger et al. 2003) Austria (Nast 1972, Holzinger et al. 2003) Belgium (Nast 1972, Holzinger et al. 2003); Canada: Alberta, British Columbia, Manitoba, New Brunswick, Newfoundland, Nova Scotia, Ontario, Quebec, Saskatchewan (Bartlett et al. 2014) Czechoslovakia (Nast 1972, Holzinger et al. 2003); Denmark (Nast 1972, Holzinger et al. 2003); Finland (Nast 1972, Holzinger et al. 2003); France (Nast 1972, Holzinger et al. 2003); Germany (Nast 1972, Holzinger et al. 2003); Great Britain (Nast 1972, Holzinger et al. 2003); Hungary (Nast 1972, Holzinger et al. 2003); Italy (Nast 1972, Holzinger et al. 2003); Japan: Hokkaido, Honshu; Macedonia; Morocco; Netherlands; Norway; Poland; Romania; Russia; Serbia; Spain; Sweden; Switzerland; Tunisia; USA: Alaska, Arizona, California, Colorado, Connecticut, Delaware, Georgia, Idaho, Illinois, Indiana, Iowa, Kansas, Maine, Maryland, Massachusetts, Michigan, Minnesota, New Hampshire, New Jersey, New Mexico, New York, North Carolina, Ohio, Oregon, Pennsylvania, Rhode Island, South Dakota, Tennessee, Utah, Vermont, Virginia, Washington, Wisconsin (Bartlett et al. 2014).

##### Notes

New record: China: Ningxia (Liupan Mountain).

#### Cixius nitobei

Matsumura, 1914

F0889B2F-2742-5663-A300-E9FDBB654A89

Cixius nitobei Matsumura, 1914: 401.| Jacobi, 1944: 14.| Schumacher, 1915: 131.

##### Distribution

China: Fujian, Taiwan (Matsumura 1914).

#### Cixius obvius

Tsaur & Hsu, 1991

2511261C-2074-50E7-817B-930B4D5E5BF3

Cixius obvius Tsaur & Hsu in Tsaur et al., 1991b: 288.

##### Distribution

China: Taiwan (Tsaur et al. 1991b).

#### Cixius operosus

Tsaur & Hsu, 1991

7398E183-14EE-568A-AD6D-8ECAF23D8133

Cixius operosus Tsaur & Hsu in Tsaur et al., 1991b: 247.

##### Distribution

China: Taiwan (Tsaur et al. 1991b).

#### Cixius parallelus

Tsaur & Hsu, 1991

9DBA15A9-AE2C-512B-8446-C5296328530E

Cixius parallelus Tsaur & Hsu in Tsaur et al., 1991b: 251.

##### Distribution

China: Taiwan (Tsaur et al. 1991b).

#### Cixius paucus

Tsaur & Hsu, 1991

5C546A07-EA28-520F-9D6F-DB41933C8241

Cixius paucus Tsaur & Hsu in Tsaur et al., 1991b: 289.

##### Distribution

China: Taiwan (Tsaur et al. 1991b).

#### Cixius perexiguus

Tsaur & Hsu, 1991

A9F3EF34-8C55-57F7-837E-048ECAE90CE4

Cixius perexiguus Tsaur & Hsu in Tsaur et al., 1991b: 236.

##### Distribution

China: Taiwan (Tsaur et al. 1991b).

#### Cixius perpendicularis

Tsaur & Hsu, 1991

F8E37213-BD8E-5E3B-B29D-59DC1D92658E

Cixius perpendicularis Tsaur & Hsu in Tsaur et al., 1991b: 291.

##### Distribution

China: Taiwan (Tsaur et al. 1991b).

#### Cixius petilus

Tsaur & Hsu, 1991

57EB871F-C6FE-5514-8D75-01A15D228886

Cixius petilus Tsaur & Hsu in Tsaur et al., 1991b: 231.

##### Distribution

China: Taiwan (Tsaur et al. 1991b).

#### Cixius phonascus

Fennah, 1956

FBE194C4-3DA8-5C39-AD5E-DAC6831CD4D2

Cixius phonascus Fennah, 1956: 449 (Fennah 1956).

##### Distribution

China: Guangdong.

#### Cixius pilosellus

Matsumura, 1914

FD706C01-92B5-5A32-B396-C775FF4FC01F

Cixius pilosellus Matsumura, 1914: 405.

##### Distribution

China: Taiwan (Matsumura 1914).

#### Cixius polydentis

Tsaur & Hsu, 1991

5CFC8CEB-CF69-504C-9E56-609AE12A510A

Cixius polydentis Tsaur & Hsu in Tsaur et al., 1991b: 297.

##### Distribution

China: Taiwan (Tsaur et al. 1991b).

#### Cixius privus

Tsaur & Hsu, 1991

F6EA01D5-3227-5A05-95B1-37C7AFACD5A7

Cixius privus Tsaur & Hsu in Tsaur et al., 1991b: 244.

##### Distribution

China: Taiwan (Tsaur et al. 1991b).

#### Cixius procerus

Tsaur & Hsu, 1991

C103796E-3F3E-534F-870A-173919101B05

Cixius procerus Tsaur & Hsu in Tsaur et al., 1991b: 278.

##### Distribution

China: Taiwan (Tsaur et al. 1991b).

#### Cixius quinarius

Tsaur & Hsu, 1991

0CC83693-5BC7-5A1D-82C0-64D01DD9F810

Cixius quinarius Tsaur & Hsu in Tsaur et al., 1991b: 249.

##### Distribution

China: Taiwan (Tsaur et al. 1991b).

#### Cixius rarus

Tsaur & Hsu, 1991

B6A7A21A-10FA-5439-A136-FBA8976DE0C3

Cixius rarus Tsaur & Hsu in Tsaur et al., 1991b: 190.

##### Distribution

China: Taiwan (Tsaur et al. 1991b).

#### Cixius reversus

Tsaur & Hsu, 1991

07F68428-C60D-5D7C-81A5-019B21D3D1E2

Cixius reversus Tsaur & Hsu in Tsaur et al., 1991b: 260.

##### Distribution

China: Taiwan (Tsaur et al. 1991b).

#### Cixius scrupeus

Fennah, 1956

B2F27C94-90D0-5E9A-894A-5FAA1A03B8F0

Cixius scrupeus Fennah, 1956: 450.| Tsaur et al., 1991b: 297.

##### Distribution

China: Anhui (Fennah 1956), Hunan, Henan, Taiwan (Tsaur et al. 1991b).

##### Notes

New record: China: Hunan (Mang Mountain), Hunan (Huping Mountain), Anhui (Guniujiang), Henan (Yuhuang).

#### Cixius segregatus

Tsaur & Hsu, 1991

70B2127E-7F19-5BA3-B763-BA105F36BA0D

Cixius segregatus Tsaur & Hsu in Tsaur et al., 1991b: 263.

##### Distribution

China: Taiwan (Tsaur et al. 1991b).

#### Cixius separatus

Tsaur & Hsu, 1991

4C534B50-5317-589D-BBCA-EE3D92BA53D7

Cixius separatus Tsaur & Hsu in Tsaur et al., 1991b: 214.

##### Distribution

China: Taiwan (Tsaur et al. 1991b).

#### Cixius serratus

Tsaur & Hsu, 1991

068325B8-EDA2-5799-975A-A78D3DE69D34

Cixius serratus Tsaur & Hsu in Tsaur et al., 1991b: 266.

##### Distribution

China: Taiwan (Tsaur et al. 1991b).

#### Cixius spinosus

Tsaur & Hsu, 1991

2F84A3BE-1C71-5194-8AAC-93E5FDB9BE6E

Cixius spinosus Tsaur & Hsu in Tsaur et al., 1991b: 282.

##### Distribution

China: Taiwan (Tsaur et al. 1991b).

#### Cixius spirus

Tsaur & Hsu, 1991

7D971480-F68E-522E-BE46-BC7682B9B33D

Cixius spirus Tsaur & Hsu in Tsaur et al., 1991b: 186.

##### Distribution

China: Taiwan (Tsaur et al. 1991b).

#### Cixius stallei

Tsaur & Hsu, 1991

F783E185-CD78-53EA-ABE8-05727E62899B

Cixius stallei Tsaur & Hsu in Tsaur et al., 1991b: 291.

##### Distribution

China: Taiwan (Tsaur et al. 1991b).

#### Cixius suturalis

Matsumura, 1914

3F44CC3C-0E14-5A3A-B66B-EA6ADC32E02C

Cixius suturalis Matsumura, 1914: 401| Tsaur et al., 1991b: 301.

##### Distribution

China: Taiwan (Matsumura 1914).

#### Cixius taipingshanus

Tsaur & Hsu, 1991

C8C3354F-16C3-5AF8-A0C8-F4D573F670EC

Cixius taipingshanus Tsaur & Hsu in Tsaur et al., 1991b: 275.

##### Distribution

China: Taiwan (Tsaur et al. 1991b).

#### Cixius taiwanus

Tsaur & Hsu, 1991

D1C2D33F-CC41-5773-99B1-FBB5A94FED80

Cixius taiwanus Tsaur & Hsu in Tsaur et al., 1991b: 294.

##### Distribution

China: Taiwan (Tsaur et al. 1991b).

#### Cixius tappanus

Matsumura, 1914

8D1A7769-442F-542A-9C85-201B110E22D2

Cixius tappanus Matsumura, 1914: 398| Tsaur et al., 1991b: 195.

##### Distribution

China: Zhejiang, Taiwan (Tsaur et al. 1991b).

##### Notes

New record: China: Zhejiang (Longwang Mountain).

#### Cixius transversus

Tsaur & Hsu, 1991

E9E2B57C-FCBB-5246-8997-706E4B713A11

Cixius transversus Tsaur & Hsu in Tsaur et al., 1991b: 229.

##### Distribution

China: Taiwan (Tsaur et al. 1991b).

#### Cixius tsuifenghuensis

Tsaur & Hsu, 1991

04D99A90-775B-546C-B4E9-2B35692C02B8

Cixius tsuifenghuensis Tsaur & Hsu in Tsaur et al., 1991b: 259.

##### Distribution

China: Taiwan (Tsaur et al. 1991b).

#### Cixius tungpuus

Tsaur & Hsu, 1991

3D74EBD9-A204-5366-9385-87016D03EF23

Cixius tungpuus Tsaur & Hsu in Tsaur et al., 1991b: 233.

##### Distribution

China: Taiwan (Tsaur et al. 1991b).

#### Cixius tzuenus

Tsaur & Hsu, 1991

CC9E81AE-9B36-5E27-B2DD-F27E88EF571D

Cixius tzuenus Tsaur & Hsu in Tsaur et al., 1991b: 222.

##### Distribution

China: Taiwan (Tsaur et al. 1991b).

#### Cixius vatius

Tsaur & Hsu, 1991

F86779CF-03FD-5E31-834E-8EB8FF5B55B5

Cixius vatius Tsaur & Hsu in Tsaur et al., 1991b: 216.

##### Distribution

China: Taiwan (Tsaur et al. 1991b).

#### Cixius velox

Matsumura, 1914

AE8D9C25-4371-5ED4-B770-0680DE58F73D

Cixius velox Matsumura, 1914: 403.

##### Distribution

China: Taiwan (Matsumura 1914).

#### Cixius vittatus

Tsaur & Hsu, 1991

15330322-B680-518B-B487-48CB2CAA6E43

Cixius vittatus Tsaur & Hsu in Tsaur et al., 1991b: 211.

##### Distribution

China: Guangxi, Ningxia, Taiwan (Tsaur et al. 1991b).

##### Notes

New record: China: Ningxia (Liupan Mountain), Guangxi (Huaping nature reserve).

#### Cixius wui

Tsaur & Hsu, 1991

47BD70F6-E135-551E-AF21-A4C0ACFB600B

Cixius wui Tsaur & Hsu in Tsaur et al., 1991b: 178.

##### Distribution

China: Taiwan (Tsaur et al. 1991b).

#### Cixius wusheus

Tsaur & Hsu, 1991

48F55DD6-70F0-5F83-BFBA-C33DAF1692A3

Cixius wusheus Tsaur & Hsu in Tsaur et al., 1991b: 269.

##### Distribution

China: Taiwan (Tsaur et al. 1991b).

#### Cixius yangi

Tsaur & Hsu, 1991

33CBE7AA-FA8E-5976-802E-2CFBD86C2EE2

Cixius yangi Tsaur & Hsu in Tsaur et al., 1991b: 192.

##### Distribution

China: Taiwan (Tsaur et al. 1991b).

#### Acanthocixius

Wagner, 1939

EF5165FF-EC18-529B-94FE-969DA492BDF4

#### Cixius (Acanthocixius) stigmaticus

(Germar, 1818)

9F418B54-257A-5A00-9884-D6DEE1614160

Flata stigmaticus Germar, 1818: 199.| *Cixiusstigmaticus* (Germar, 1818), Stephens, 1829: 357.| Cixius (Acanthocixius) stigmaticus (Germar, 1818), Mozaffarian & Wilson, 2011: 9.| *Cixiusstigmaticus* (Germar, 1818), Emeljanov, 2015: 115.

##### Distribution

China: Guangxi, Guizhou, Zhejiang; France (Ribaut and Lacroix 1958); Germany (Holzinger et al. 2003); UK (Holzinger et al. 2003); Iran: Kandovān (Mozaffarian and Wilson 2011); Netherlands (De Haas and Den Bieman 2018); Poland (Gebicki et al. 2013).

##### Notes

New record: China: Guangxi (Huaping), Zhejiang (Hangzhou).

#### Ceratocixius

Wagner, 1939

90E3DCFE-59E7-563B-9F31-4679FF4777BE

#### Cixius (Ceratocixius) subsimplex

Vilbaste, 1968

8A5193E5-1BC3-51A2-B887-65D3CA2783E5

Cixius subsimplex Vilbaste, 1968: 5.| Anufriev & Emeljanov, 1988: 452.| Cixius (Ceratocixius) subsimplex, Emeljanov, 2015: 98.

##### Distribution

China: Gansu.

##### Notes

New record: China: Gansu (Wenxian).

#### Gonophallus

Tsaur & Hsu, 1991

3A91091B-82C9-57B2-B78E-601D14BEAD36

#### Gonophallus trinus

Tsaur & Hsu, 1991

DC6F69CE-D1AB-59FE-B628-1806A268ED1D

Gonophallus trinus Tsaur & Hsu in Tsaur et al., 1991a: 25.

##### Distribution

China: Taiwan(Tsaur et al. 1991a).

#### Macrocixius

Matsumura, 1914

8EBE9C65-309F-5F97-B3F5-04E1C963CCAE

#### Macrocixius giganteus

Matsumura, 1914

7965E741-4BE1-5D63-8869-9FE072989844

Macrocixius giganteus Matsumura, 1914: 394.| Schumacher, 1915: 131.| Fennah, 1956: 459.| Tsaur et al., 1991a: 3.| Liang, 2005: 429.| Orosz, 2013: 107.| Zhang & Chen, 2013b: 279.| Hayashi & Fujinuma, 2016: 325.

##### Distribution

China: Fujian, Hainan, Jiangxi (Zhang and Chen 2013a), Taiwan (Tsaur et al. 1991a); Japan: Kyushu; Vietnam (Hayashi and Fujinuma 2016).

##### Notes

New record: China: Fujian (Chongan), Hainan (Jianfeng), Jiangxi (Wuyishan).

#### Macrocixius grossus

Tsaur & Hsu, 1991

62A20272-C6C5-5F98-9370-4D8287C0D5CB

Macrocixius grossus Tsaur & Hsu in Tsaur et al. 1991a: 5.| Orosz, 2013: 108.| Zhang & Chen, 2013b: 281.

##### Distribution

China: Guizhou, Sichuan, Yunnan, Zhejiang (Zhang and Chen 2013a), Taiwan (Tsaur et al. 1991a); Vietnam.

##### Notes

New record: China: Guizhou (Luodian).

#### Macrocixius rarimaculatus

Zhang & Chen, 2013

6FF32F2F-2935-581A-89F6-9629F8731AAD

Macrocixius rarimaculatus Zhang & Chen, 2013a: 283.| Orosz & Redei, 2016: 376.

##### Distribution

China: Guizhou (Zhang and Chen 2013a), Jiangxi, Taiwan (Tsaur et al. 1991a); Nepal: Ganesh Himal.

#### Macrocixius unispinus

Zhang & Chen, 2013

917E8EAC-3D2A-54DD-8EB8-73EFECA23149

Macrocixius unispinus Zhang & Chen, 2013a: 285.

##### Distribution

China: Yunnan (Zhang and Chen 2013a) .

#### Semicixius

Tsaur & Hsu, 1991

04CF99CF-C06D-5829-A7EE-174190280F3E

#### Semicixius denticulus

Tsaur & Hsu, 1991

E46700BA-100D-56EE-AE64-4DAE93EF1327

Semicixius denticulus Tsaur & Hsu in Tsaur et al., 1991a: 23.

##### Distribution

China: Taiwan (Tsaur et al. 1991a).

#### Tsauria

Kocak & Kemal, 2009

7875DF93-D0AE-5BDC-9661-20FB930ED5F1

#### Tsauria brevispina

Zhi & Chen, 2019

60BE95E6-7AFB-5A3A-AC97-F85272C0DDC3

Tsauria brevispina Zhi & Chen, 2019: 57.

##### Distribution

China: Guizhou, Hubei (Zhi et al. 2019).

#### Tsauria cehengensis

(Zhang & Chen, 2011)

D9DDA4B9-2F07-5C23-8FF5-EE3C8EC609E0

Discophorellus cehengensis Zhang & Chen, 2011a: 61.| *Tsauriachengensis* (Zhang & Chen, 2011), Xing, 2014: 149.

##### Distribution

China: Guizhou (Zhang and Chen 2011a).

#### Tsauria longispina

Zhi & Chen, 2019

7ED7A33A-AFC8-57BF-8BCB-5E77C5E4EABC

Tsauria longispina Zhi & Chen, 2019: 63.

##### Distribution

China: Fujian, Guizhou, Hainan (Zhi et al. 2019).

#### Tsauria major

(Tsaur & Hsu, 1991)

E5961B02-F107-507A-8A23-8CE454C1DB4F

Discophorellus major Tsaur & Hsu in Tsaur et al., 1991a: 21.| *Tsauriamajor* (Tsaur & Hsu, 1991), Kocak, 2009: 6.

##### Distribution

China: Taiwan (Tsaur et al. 1991a).

#### Tsauria transspinus

(Zhang & Chen, 2011)

67C9833E-7F1F-56CF-B2F4-C0367692FBAB

Discophorellus transspinus Zhang & Chen, 2011a: 64.| *Tsauriatransspinus* (Zhang & Chen, 2011), Xing, 2014: 150.

##### Distribution

China: Guizhou (Zhang and Chen 2011a).

#### Eucarpiini

Emeljanov, 2002

2A3FAE39-9A21-5D85-9A15-2BAFE85FA909

#### Bajauana

Distant, 1907

375B3D3B-998D-5604-9709-844CFEC72F28

#### Bajauana mestra

Fennah, 1980

0EA3DB4A-E064-5804-B9FE-E6E4B7F31DB0

Bajauana mestra Fennah, 1980: 285.

##### Distribution

China: Hunan; Indonesia: Irian Jaya (Fennah 1980).

##### Notes

New record: China: Hunan (Nanyue).

#### Bajauana smaragus

Fennah, 1980

666BFF28-7E87-5850-953F-FF7AA90A01EA

Bajauana smaragus Fennah, 1980: 277.

##### Distribution

China: Hainan; Indonesia: Irian Jaya (Fennah 1980).

##### Notes

New record: China: Hainan (Qixianling).

#### Dilacreon

Fennah, 1980

F090A2EC-A24A-504F-8D6B-DCD39A3E378D

#### Dilacreon

Fennah, 1980

5C21B03C-3AF1-5E30-AE09-BE39E0E33A2B

#### Dilacreon (Dilacreon) semiramis

Fennah, 1980

1CEF4E28-E1DB-590E-BB73-873D36C862B4

Dilacreon (Dilacreon) semiramis Fennah, 1980: 242.

##### Distribution

China: Hainan; Indonesia: Irian Jaya; Papua New Guinea: Hollandia (Fennah 1980).

##### Notes

New record: China: Hainan (Wuzhi Mountain).

#### Eucarpia

Walker, 1857

AFFFE019-AB44-56D2-9646-F6BFE257086D

#### Eucarpia specialis

Tsaur & Hsu, 2003

F9B5B7B3-CFA3-55DE-B06A-715A4BE0F2D2

Eucarpia specialis Tsaur & Hsu, 2003: 438.

##### Distribution

China: Hainan, Taiwan (Tsaur and Hsu 2003).

##### Notes

New record: China: Hainan (Wuzhi Mountain).

#### Eucarpia stellata

Tsaur & Hsu, 2003

71278946-0526-5FDD-97FC-AC27301330C9

Eucarpia stellata Tsaur & Hsu, 2003: 436.

##### Distribution

China: Fujian, Hainan, Taiwan (Tsaur and Hsu 2003).

##### Notes

New record: China: Hainan (Liping), Fujian (Meihua).

#### Eucarpia truncata

Tsaur & Hsu, 2003

07061F9B-7191-506E-98CC-48873A2FBD41

Eucarpia truncata Tsaur & Hsu, 2003: 438.

##### Distribution

China: Taiwan (Tsaur and Hsu 2003).

#### Ptoleria indica

(Distant, 1916)

449AF2BE-4A78-5E53-A9FA-CEDAF4182C0A

Ptoleria indica ,*Caneironaindica* Distant, 1916: 39.| *Ptoleriaindica* (Distant, 1916), Fennah, 1956: 448.

##### Distribution

China: Hubei (Fennah 1956); India (Distant 1916).

##### Notes

This species is recorded here from China based on female specimens of literature data.

#### Kirbyana

Distant, 1906

BCA41184-DFC7-5312-AB2A-7E06F895EDC6

#### Kirbyana aspina

Zhi & Chen, 2021

C9548C7A-58F9-5DBF-879B-336E7471F61E

Kirbyana aspina Zhi & Chen in Zhi et al., 2021: 7.

##### Distribution

China: Hunan (Zhi et al. 2021).

#### Kirbyana furcata

Zhi & Chen, 2021

854B6BD6-ECA4-57DC-9FF0-DC679DC0C083

Kirbyana furcata Zhi & Chen in Zhi et al., 2021: 8.

##### Distribution

China: Yunnan, Guangxi (Zhi et al. 2021).

#### Kirbyana lini

Tsaur & Hsu, 2003

0B1AF926-9B85-5181-9CD2-5E335AB7203C

Kirbyana lini Tsaur & Hsu, 2003: 434.

##### Distribution

China: Taiwan (Tsaur and Hsu 2003).

#### Kirbyana pagana

(Melichar, 1903)

D327AD6A-371B-5E51-8F93-D81A71AE0B61

Kirby pagana Melichar, 1903: 248.| *Kirbyanapagana* (Melichar, 1903), Distant, 1907: 262.| Tsaur, 2003: 432.

##### Distribution

China: Hainan, Taiwan (Tsaur and Hsu 2003); India; Malaysia; Sri Lanka: Peradeniya.

##### Notes

New record: China: Hainan (Wuzhi Mountain).

#### Neocarpia

Tsaur & Hsu, 2003

6AB025AB-3F20-5337-A8CA-F3A4D8654124

#### Neocarpia acutata

Zhi & Chen, 2017

66424E8C-DAA6-5CC3-B4D5-01F049F4DE27

Neocarpia acutata Zhi & Chen in Zhi et al., 2017: 23.

##### Distribution

China: Yunnan (Zhi et al. 2017).

#### Neocarpia bidentata

Zhang & Chen, 2013

8C795E2B-10E6-5F7D-9DCD-03DF6717D698

Neocarpia bidentata Zhang & Chen, 2013b: 43.

##### Distribution

China: Guizhou (Zhang and Chen 2013b).

#### Neocarpia hamata

Zhang & Chen, 2013

72A1DA6E-2AC6-5C85-9606-A8C0144C54B5

Neocarpia hamata Zhang & Chen, 2013b: 45.| Zhi et al., 2017: 27.

##### Distribution

China: Guizhou, Hubei (Zhang and Chen 2013b).

#### Neocarpia maai

Tsaur & Hsu, 2003

F8C7F407-C432-5D45-B21A-62EB85C1C8A9

Neocarpia maai Tsaur & Hsu, 2003: 441.

##### Distribution

China: Zhejiang, Taiwan (Tsaur and Hsu 2003).

##### Notes

New record: China: Zhejiang (Fengyang Mountain).

#### Neocarpia reversa

Zhi & Chen, 2017

393C1576-5A2D-502F-809F-F306AE8FF485

Neocarpia reversa Zhi & Chen in Zhi et al., 2017: 30.

##### Distribution

China: Yunnan (Zhi et al. 2017).

#### Oecleini

Muir, 1922

ECDC80C1-C224-5B55-A086-ABA848F27A2A

#### Haplaxius

Fowler, 1904

1DA44414-3DA0-584C-B3F9-E496EDCE92A7

#### Haplaxius ovatus

Ball, 1933

951AB21E-6356-503C-B8CA-964D897F7FDD

Myndus ovatus Ball, 1933: 473.| *Haplaxiusovatus* (Ball, 1933), Caldwell, 1946: 203.| *Myndusovatus* (Ball, 1933), Kramer, 1979: 344.| *Haplaxiusovatus* (Ball, 1933), Emeljanov, 1989: 62.| Bartlett et al., 2014: 99| Wheeler, 2014: 360.

##### Distribution

China: Guizhou; USA: Delaware, Georgia, Illinois, Iowa, Kansas, Maryland, Massachusetts, Minnesota, Missouri, Nebraska, New Jersey, Oklahoma, South Dakota, Virginia, Wisconsin (Bartlett et al. 2014).

#### Mundopa

Distant, 1906

F516293B-AB9D-5F10-8690-4F9D81B21CB3

#### Mundopa kotoshonis

Matsumura, 1914

38AA8E5E-80E6-5A7E-B0BE-B3499FE66013

Mundopa kotoshonis Matsumura, 1914: 430.| Tsaur et al., 1991a: 76.

##### Distribution

China: Taiwan (Tsaur et al. 1991a).

#### Myndus

Stål, 1862

8BFFA4B0-4570-5363-82F2-6BC9A97EB053

#### Myndus kotoshonis

Matsumura, 1940

16C7BBAA-6755-5128-929B-D57F238DAD71

Myndus kotoshonis Matsumura, 1940: 45.| Tsaur et al., 1991a: 74.

##### Distribution

China: Taiwan (Tsaur et al. 1991a).

#### Pentastirini

Emeljanov, 1971

43228163-1349-5B53-9275-3A7159115794

#### Oteana

Hoch, 2006

DC7D75CD-D6DA-502A-A302-B82A500118CC

#### Oteana oryzae

(Matsumura, 1911)

A9DC88F3-2DF3-54E2-AD1E-ED95EE5554AE

Oliarus oryzae Matsumura, 1911: 134.| Van Stalle, 1991: 34.| *Oteanaoryzae* (Matsumura, 1911), Emeljanov, 2007: 291.

##### Distribution

China: Taiwan (Van Stalle 1991).

#### Pentastirina

Emeljanov, 1971

0A15774F-4A72-586F-9261-EBA8354EC7B6

#### Arosinus

Emeljanov, 2007

D90C93E3-6028-5FAA-8D6D-574DBDAE7D48

#### Arosinus hopponis

(Matsumura, 1914)

6FC9E43C-C7FB-5686-912C-3864478B3584

Oliarus boninensis Matsumura, 1914: 423.| Fennah, 1956: 83; Van Stalle, 1991: 46.| *Arosinusboninensis* (Matsumura, 1914), Emeljanov, 2007: 291.| Hayashi & Fujinuma, 2016: 323.

##### Distribution

China: Taiwan (Matsumura 1914).

#### Arosinus velox

(Matsumura, 1914)

F02EDA15-7BC1-5235-A6FE-063D9578F20A

Oliarus velox Matsumura, 1914: 425.| *Arosinusvelox* (Matsumura, 1914), Emeljanov, 2007: 291.

##### Distribution

China: Taiwan (Matsumura 1914).

#### Atretus

Emeljanov, 2007

9B34F8EC-CFE0-52E5-9C13-27A3407A6273

#### Atretus horishanus

(Matsumura, 1914)

DE0DF89B-7483-5166-B984-40C68D9B9756

Oliarus horishanus Matsumura, 1914: 418.| Schumacher, 1915: 131.| Van Stalle, 1991: 84.| Liang, 2005: 429.| *Atretushorishanus* (Matsumura, 1914), Emeljanov, 2007: 291.

##### Distribution

China: Taiwan (Matsumura 1914).

#### Atretus hsui

(Tsaur, 1990)

DFE1B721-7C54-5D8C-B3BB-CBBD3F14FEC9

Oliarus hsui Tsaur, 1990b: 135.| *Atretushsui* (Tsaur, 1990), Emeljanov, 2007: 291.

##### Distribution

China: Taiwan (Tsaur 1990b).

#### Atretus nigronervatus

(Fennah, 1956)

3BFB83F3-7205-5FBD-9A7C-5E129C08702E

Oliarus nigronervatus Fennah, 1956: 451.| Liang, 2005a: 429.| *Atretusnigronervatus* (Fennah, 1956), Emeljanov, 2007: 291.

##### Distribution

China: Fujian, Guangxi, Hubei (Fennah 1956).

##### Notes

New record: China: Guangxi (Baiyangsi).

#### Atretus shiaoi

(Tsaur, 1990)

FC8BF61C-9D3C-50D4-98FD-1B404BABFFB5

Oliarus shiaoi Tsaur, 1990b: 137.| *Atretusshiaoi* (Tsaur, 1990), Emeljanov, 2007: 291.

##### Distribution

China: Taiwan (Tsaur 1990b).

#### Atretus yangi

(Tsaur, 1989)

490B69BF-73DD-5472-AA66-4AAC9429CEBB

Oliarus yangi Tsaur, 1989a: 171.| Van Stalle, 1991: 84.| *Atretusyangi* (Tsaur, 1989), Emeljanov, 2007: 291.

##### Distribution

China: Taiwan (Tsaur 1989a).

#### Indolipa

Emeljanov, 2001

5F512EC6-101E-5A59-980D-745D6C39C418

#### Indolipa fopingensis

Luo, Liu & Feng 2019

BECDBE51-F693-55AA-89C0-8FEBA180E387

Indolipa fopingensis Luo, Liu & Feng, 2019b: 184.

##### Distribution

China: Shaanxi (Luo et al. 2019b).

#### Indolipa longlingensis

Zhi & Chen, 2020

ABC6A230-B806-5CF5-B596-0CEF0D09A040

Indolipa fugongensis Zhi & Chen in Zhi et al., 2020b: 22.

##### Distribution

China: Yunnan (Zhi et al. 2020b).

#### Indolipa gansuensis

Guo & Feng, 2010

8C225D8A-5EBD-5AB6-9B41-12DA2EDF330D

Indolipa gansuensis Guo & Feng, 2010: 35.

##### Distribution

China: Gansu (Guo and Feng 2010).

#### Indolipa huapingensis

Luo, Liu & Feng, 2019

6B12023A-D946-596F-B2F5-F9A22D142DDB

Indolipa huapingensis Luo, Liu & Feng, 2019b: 189.

##### Distribution

China: Guangxi (Luo et al. 2019b).

#### Indolipa kurseongensis

(Distant, 1911)

C57FB780-26F4-5717-B931-CF90F3FC2AF6

Oliarus kurseongensis Distant, 1911: 737.| Fennah, 1956: 451.| Van Stalle, 1991: 51.| *Indolipakurseongensis* (Distant, 1911), Emeljanov, 2001: 72.| Guo & Feng, 2010: 38.| Luo, Liu & Feng, 2019b: 192.

##### Distribution

China: Hubei (Fennah 1956), Guangxi, Hunan, Yunnan (Luo et al. 2019b), Tibet (Guo and Feng 2010); India: Darjeeling (Van Stalle 1991).

#### Indolipa longlingensis

Zhi & Chen, 2020

9EF4334D-1F94-5A49-BF0F-E28406861B25

Indolipa longlingensis Zhi & Chen in Zhi et al., 2020b: 25.

##### Distribution

China: Yunnan (Zhi et al. 2020b).

#### Indolipa tappanus

(Matsumura, 1914)

93D7B569-FB05-5852-8017-6A76C2867264

Oliarus tappanus Matsumura, 1914: 424.| Tsaur, 1988: 46.| Van Stalle, 1991: 51.| *Indolipatappanus* (Matsumura, 1914), Emeljanov, 2001: 72.| Guo & Feng, 2010: 41.

##### Distribution

China: Hainan, Taiwan (Guo and Feng 2010).

##### Notes

New record: China: Hainan (Diaoluo Mountain).

#### Melanoliarus

Fennah, 1945

6B0A7940-7BA2-5AF9-8DE5-B31945E333C0

#### Melanoliarus canyonensis

(Mead & Kramer, 1981)

872E87FC-4E42-55A4-8500-3FDD55F99E0F

Oliarus canyonensis Mead & Kramer, 1982: 381.| *Melanoliaruscanyonensis* (Mead & Kramer, 1981), Bartlett et al., 2014: 92.

##### Distribution

China: Taiwan; Japan; USA: California, New Mexico (Bartlett et al. 2014).

##### Notes

This species is recorded here from China based on female specimens of literature data.

#### Melanoliarus vicarius

(Walker, 1851)

3FF896B8-0904-5CEA-9754-9FAAE0AE0933

Cixius vicaria Walker, 1851: 343.| *Oliarusvicarius* (Walker, 1851), Distant, 1907: 282.| *Oliaruslucidus* Metcalf, 1936: 79.|*Oliarusvicarius* (Walker, 1851), Mead & Kramer, 1981: 390.| *Melanoliarusvicarius* (Walker, 1851), Emeljanov, 2001: 122.| Bartlett et al., 2014: 96.

##### Distribution

China: Hunan; USA: Florida, Colorado; Georgia; Illinois; Maryland; Massachusetts; Mississippi; New Jersey; North Carolina; South Carolina; Texa (Bartlett et al. 2014).

##### Notes

This species is recorded here from China based on female specimens of literature data.

#### Oecleopsis

Emeljanov, 1971

372F7A3B-9248-5955-91EB-15A82C524580

#### Oecleopsis articara

Van Stalle, 1991

3837287A-9DA6-5545-A88B-E975D9FF9122

Oecleopsis articara Van Stalle, 1991: 22.| Guo et al., 2009: 48.

##### Distribution

China: Hainan, Henan, Sichuan (Guo et al. 2009), Guizhou; Malaysia: Borneo, Pahang (Van Stalle 1991).

##### Notes

New record: China: Guizhou (Duyun). This species is recorded here from China only based on female specimens.

#### Oecleopsis bifidus

(Tsaur, Hsu & Van Stalle, 1988)

714BFD67-FCB0-5DCB-8D69-E615B4F8E8C6

Oliarus bifidus Tsaur, Hsu & Van Stalle, 1988: 52.| *Oecleopsisbifidus* (Tsaur, Hsu & Van Stalle, 1988), Van Stalle, 1991: 25.| Guo et al., 2009: 48.

##### Distribution

China: Fujian, Taiwan (Tsaur et al. 1988).

##### Notes

New record: China: Fujjian (Shaowu).

#### Oecleopsis chiangi

(Tsaur, Hsu & Van Stalle, 1988)

A646C45B-654E-50C8-99D9-1919D68F09FC

Oliarus chiangi Tsaur, Hsu & Van Stalle, 1988: 50.| *Oecleopsischiangi* (Tsaur, Hsu & Van Stalle, 1988), Van Stalle, 1991: 26.| Guo et al., 2009: 49.

##### Distribution

China: Fujian, Taiwan (Tsaur et al. 1988).

##### Notes

New record: China: Fujjian (Shaowu).

#### Oecleopsis elevatus

(Tsaur, Hsu & Van Stalle, 1988)

DE2C66BA-BF7B-52EF-8784-7CD89FC3A020

Oliarus elevatus Tsaur, Hsu & Van Stalle, 1988: 53.| *Oecleopsiselevatus* (Tsaur, Hsu & Van Stalle, 1988), Van Stalle, 1991: 26.| Guo et al., 2009: 49.| Hayashi & Fujinuma, 2016: 325.

##### Distribution

China: Guangxi, Taiwan (Tsaur et al. 1988); Japan: Honshu (Hayashi and Fujinuma 2016).

##### Notes

New record: China: Guangxi (Lingchuan).

#### Oecleopsis laminatus

Zhi & Chen, 2018

6644832B-95D5-51EC-8EBA-2DC149443E2C

Oecleopsis laminatus Zhi & Chen in Zhi et al., 2018a: 5.

##### Distribution

China: Yunnan (Zhi et al. 2018a).

#### Oecleopsis mori

Matsumura, 1914

4837A9C9-CCA8-5DE7-8E41-1D7BE8F2855B

Oecleopsis mori Matsumura, 1914: 426.| Van Stalle, 1991: 23.| Guo et al., 2009: 50.| Zhi et al., 2018a: 9.

##### Distribution

China: Guangxi, Yunnan (Zhi et al. 2018a), Taiwan (Van Stalle 1991).

#### Oecleopsis petasatus

(Noualhier, 1896)

C6F3EA86-F66C-5F35-B4A7-237610233A31

Oliarus petasatus Noualhier, 1896: 255.| Fennah, 1956: 455.| *Oecleopsispetasatus* (Noualhier, 1896), Van Stalle, 1991: 22.| Guo et al., 2009: 50.

##### Distribution

China: Hainan, Sichuan, Yunnan (Guo et al. 2009); Cambodia: (Noualhier 1896).

##### Notes

New record: China: Yunnan (Yaoqu, Mengla, Longling, Kunming), Sichuan (Yaan), Hainan (Yinggeling).

#### Oecleopsis productus

Zhi & Chen, 2018

BF059895-42F7-5A75-A592-AD29073A89B7

Oecleopsis productus Zhi & Chen in Zhi et al., 2018a: 9.

##### Distribution

China: Yunnan (Zhi et al. 2018a).

#### Oecleopsis sinicus

(Jacobi, 1944)

CC5D8363-DCC2-5B5F-9C73-831542D62556

Mnemosyne sinica Jacobi, 1944: 12.| Chou, 1985: 23.|*Oliarussinicus* (Jacobi, 1944), Van Stalle, 1988: 46.| *Oecleopsissinicus* (Jacobi, 1944), Van Stalle, 1991: 23.| Liang, 2005b: 429.| Guo et al., 2009: 45.| Hayashi & Fujinuma, 2016: 325.

##### Distribution

China: Beijing, Anhui, Fujian (Jacobi 1944), Guangdong, Guangxi (Guo et al. 2009), Henan, Hubei, Hunan, Sichuan, Guizhou, Zhejiang, Taiwan; Cambodia; Japan: Kyushu (Hayashi and Fujinuma 2016).

##### Notes

New record: China: Beijing (Mentougou), Hunan (Chenzhou, Huping), Fujian (Fuzhou), Guangxi (Lingchuan), Guangdong (Lohchan).

#### Oecleopsis spinosus

Guo & Wang, 2009

622FEAE2-33D8-5D55-8FA4-D4B576743D34

Oecleopsis spinosus Guo & Wang in Guo et al., 2009: 54.

##### Distribution

China: Shaanxi (Guo et al. 2009).

#### Oecleopsis tiantaiensis

Guo & Wang, 2009

8ED00389-A5E0-5283-8807-61F71C258082

Oecleopsis tiantaiensis Guo & Wang in Guo et al., 2009: 54.

##### Distribution

China: Shaanxi (Guo et al. 2009), Gansu.

##### Notes

New record: China: Shaanxi (Hanzhong), Gansu (Xiaolong Mountain).

#### Oecleopsis wuyiensis

Guo & Wang, 2009

A6ABBBED-2B4B-5EF6-92FA-FD44E56AFF81

Oecleopsis wuyiensis Guo & Wang in Guo et al., 2009: 56.

##### Distribution

China: Fujian, Shaanxi, Henan, Hunan (Guo et al. 2009), Yunnan.

##### Notes

New record: China: Yunnan (Lvchun).

#### Oecleopsis yoshikawai

(Ishihara, 1961)

650F8AC3-4E94-5444-8D65-A768BFB7A164

Oliarus yoshikawai Ishihara, 1961: 228.| *Oecleopsisyoshikawai* (Ishihara, 1961), Van Stalle, 1991: 22.| Guo et al., 2009: 58.| Zhi et al., 2018a: 12.

##### Distribution

China: Guizhou (Zhi et al. 2018a), Yunnan; Thailand: Doi Inthanon (Van Stalle 1991).

##### Notes

New record: China: Yunnan (Sumie).

#### Oecleopsis cucullatus

(Noualhier, 1896) comb. nov.

9EB60169-8829-5994-B4C1-DCAA23ECF8A2

Oliarus cucullatus Noualhier, 1896: 255.| Jacobi, 1917: 11.| Fennah, 1956: 453.| *Oecleuscucullatus* (Noualhier, 1896), Emeljanov, 1971: 621.

##### Distribution

China: Guangdong, Hubei (Fennah 1956); Cambodia (Van Stalle 1991).

##### Notes

This species was originally belonged to *Oecleus*, and when the authors observed the paratype specimens of this species, we found that its morphology indicates the misclassification of this species, this species with strongly elevated and foliaceous lateral carinae, consistent with the diagnostic characteristics of *Oecleopsis*, so in this study this species was transferred to *Oecleopsis* as a new combination.

#### Oliarus

Stål, 1862

DBB9B0D5-FB48-5A5E-99BA-124908EBC624

#### Oliarus bizonatus

Kato, 1932

A0D67427-A39E-5EDC-A723-9788E1C22167

Oliarus bizonatus Kato, 1932: 216.

##### Distribution

China: Northwestern of China (Kato 1932).

#### Oliarus cingalensis

(Distant, 1911)

6DD142DE-48F5-5F91-BC0F-97CB3FAB9F78

Mnemosyne cingalensis Distant, 1911: 738.| *Oliaruscingalensis* (Distant, 1911), Van Stalle, 1988: 46.| Van Stalle, 1991: 82.

##### Distribution

China: Yunnan; Sri Lanka: Trincomalee (Distant 1911); USA: Puerto Rico.

##### Notes

New record: China: Yunnan (Yuanmou).

#### Oliarus indicus

Distant, 1911

E82674FB-FEFC-587D-A5B9-4793DCD9B350

Oliarus indicus Distant, 1911: 735.| Van Stalle, 1991: 80.

##### Distribution

China: Beijing; India: (Van Stalle 1991).

##### Notes

New record: China: Beijing (Xishan).

#### Oliarus mlanjensis

Van Stalle, 1987

60E32BE8-7062-5E1C-B751-9C3F1C74D968

Oliarus mlanjensis Van Stalle, 1987: 66.

##### Distribution

China: Guangxi, Hubei; Malawi; Tanzania; Zimbabwe: ex Rhodesia (Van Stalle 1987).

##### Notes

New record: China: Guangxi (Longsheng), Hubei (Shennongjia).

#### Oliarus speciosus

Matsumura, 1914

8B86B655-1939-5F8C-8051-0BF8D984A1BE

Oliarus speciosus Matsumura, 1914: 424.

##### Distribution

China: Taiwan (Matsumura 1914).

#### Oliarus zaoensis

Wang, 1991

DCCAD982-D5BC-5594-B2BE-DD07C6A07D2C

Oliarus zaoensis Wang, 1991: 85.

##### Distribution

China: Hebei (Wang 1991).

#### Oliparisca

Emeljanov, 2001

F0C80414-8BE8-5C6E-A80B-E117D88E93FB

#### Oliparisca pundaloyensis

(Van Stalle, 1991)

F9E92CB3-0565-5575-A2F5-F4CC4BA6BADB

Oliarus pundaloyensis Van Stalle, 1991: 72.|*Olipariscapundaloyensis* (Van Stalle, 1991), Emeljanov, 2001: 72.

##### Distribution

China: Tibet; Sri Lanka: (Van Stalle 1991).

#### Pentastiridius

Kirschbaum, 1868

CB5C33E1-8FA5-51B9-9B4A-AAF5961A5EB9

#### Pentastiridius apicalis

(Uhler, 1896)

8F546EFF-29BA-524A-B1A2-D8575630C03A

Myndus apicalis Uhler, 1896: 281.| *Oliarusapicalis* (Uhler, 1896), Matsumura, 1900: ?.| Chou, 1985: 21.| *Pentastiridiusapicalis* (Uhler, 1896), Emeljanov, 1979: 223.| Anufriev & Emeljanov, 1988: 463.| Van Stalle, 1991: 15.| Liang, 2005b: 429.| Anufriev, 2009: 68.| Hayashi & Fujinuma, 2016: 326.

##### Distribution

China: Beijing (Liang 2005b), Shanghai, Fujian, Jiangxi, Jiangsu, Shaanxi, Sichuan, Zhejiang; Japan: Hokkaido, Honshu, Kyushu, Shikoku (Hayashi and Fujinuma 2016); Russia: Khabarovsk, Primorye.

##### Notes

New record: China: Hebei (Shijiazhuang), Jiangsu (Jinshan), Shanghai (Songjiang).

#### Pentastiridius bohemani

(Stål, 1859)

76A00D13-EC3F-53E9-9C38-FC4F58348173

Cixius bohemani Stål, 1859: 272.| *Oliarusbohemani* (Stål, 1859), Stål, 1862: 306.| *Pentastiridiusbohemani* (Stål, 1895), Van Stalle, 1991: 12.

##### Distribution

China: Hainan, Hongkong (Van Stalle 1991).

##### Notes

New record: China: Hainan (Xisha).

#### Pentastiridius leporinus

(Linné, 1761)

5AE3C81D-91CF-5BA8-801C-35B67142779F

Cicada leporinus Linné, 1761: 242.| *Cixiusleporinus* (Linné, 1761), Curtis, 1829: 194.| *Flataleporina* (Linné, 1761), Germar, 1830: 50.| *Oliarusleporinus* (Linné, 1761), Scott, 1870: 720.| *Pentastiridiusleporinus* (Linné, 1761), Van Stalle, 1985: 441.| Kalkandelen, 1990: 3.

##### Distribution

China: Nei-Mongol, Heilongjiang; Iran: bādeh, Albāji, Bampur, Bazmān, Birjand, Chābahār, Dālaki, Evin, Gambuyeh, Gāvbandi, Gharechaman, Hafttappeh, Hamidieh, Hāresābād, Hashtpar, Irānshahr, Kandovān (Māzandarān), Marand, Miāneh-ZanjānRd, Minushahr, Mollāsāni, Shādegān, Shieh, Susangerd, Suza, Tabriz, Varāmin, Zābol (Kalkandelen 1990); Afghanistan (Nast 1972, Holzinger et al. 2003); Albania; Algeria: (Nast 1972, Holzinger et al. 2003) Armenia; Austria; Azerbaijan (Nast 1972, Holzinger et al. 2003); Belgium: (Nast 1972, Holzinger et al. 2003); Cyprus; Czech Republic; Denmark: (Nast 1972, Holzinger et al. 2003); Estonia: (Nast 1972, Holzinger et al. 2003); Finland: (Nast 1972, Holzinger et al. 2003); France: (Nast 1972, Holzinger et al. 2003); Georgia: (Nast 1972, Holzinger et al. 2003); Germany: (Nast 1972, Holzinger et al. 2003); UK: (Nast 1972, Holzinger et al. 2003); Greece: (Nast 1972, Holzinger et al. 2003); Hungary: (Nast 1972, Holzinger et al. 2003); Ireland: (Nast 1972, Holzinger et al. 2003); Israel: (Nast 1972, Holzinger et al. 2003); Italy: (Nast 1972, Holzinger et al. 2003); Jordan: (Nast 1972); Kazakhstan: (Holzinger et al. 2003); Kyrgyzstan: (Nast 1972, Holzinger et al. 2003); Libya: (Nast 1972); Lithuania; Moldova: (Holzinger et al. 2003); Mongolia; Netherlands: (Nast 1972, Holzinger et al. 2003); Poland: (Nast 1972, Holzinger et al. 2003); Portugal: (Nast 1972, Holzinger et al. 2003); Romania: (Nast 1972, Holzinger et al. 2003); Russia: Primorye; Slovakia; Spain; Sweden; Switzerland; Tadzhikistan: (Nast 1972, Holzinger et al. 2003); Tunisia (Nast 1972); Turkey (Kalkandelen 1990); Turkmenistan (Kalkandelen 1990); Ukraine: (Nast 1972, Holzinger et al. 2003); Yugoslavia:(Nast 1972, Holzinger et al. 2003).

#### Pentastiridius pachyceps

(Matsumura, 1914)

048A3ED1-DB16-5E75-831A-96563D02A93D

Oliarus pachyceps Matsumura, 1914: 420.| Schumacher, 1915: 131.| *Pentastiridiuspachyceps* (Matsumura, 1914), Van Stalle, 1991: 13.| Hayashi & Fujinuma, 2016: 326.

##### Distribution

China: Hainan, Taiwan (Van Stalle 1991); Nansei-shoto: Ryukyu Islands (Hayashi and Fujinuma 2016).

##### Notes

New record: China: Hainan (Lingshui).

#### Pentastiridius tsoui

(Muir, 1925)

5FF2ECDC-4A01-5509-A6C1-38A4C3CE5940

Oliarus tsoui Muir, 1925: 365.| *Nesopompetsoui* (Muir, 1925), Fennah, 1956: 455.| *Pentastiridiustsoui* (Muir, 1925), Van Stalle, 1991: 16.

##### Distribution

China: Jiangsu, Hubei (Fennah 1956); Japan (Fennah 1956).

#### Reptalus

Emeljanov, 1971

14B256F2-310B-5756-8933-08AE993BB69B

#### Reptalus arcbogdulus

(Dlabola, 1985)

F38B23E2-F600-55B7-9500-A5CD0477D3BE

Oliarus arcbogdulus Dlabola, 1965: 87.| *Reptalusarcbogdulus* (Dlabola, 1965), Emeljanov, 1971: 622.| Emeljanov, 1982: 111.| Emeljanov, 2015: 209.

##### Distribution

China: Beijing; Mongolia: Uburchangaj aimak (Anonymous 2015).

##### Notes

New record: China: Beijing (Mentougou).

#### Reptalus basiprocessus

Guo & Wang, 2007

0B88D95B-EDDA-5457-8ED2-119DDABFA6FE

Reptalus basiprocessus Guo & Wang, 2007: 276.| Bai et al., 2015: 37.

##### Distribution

China: Fujian (Bai et al. 2015), Hubei, Hunan (Guo and Wang 2007), Zhejiang, Sichuan, Guizhou, Hebei, Qinghai, Jiangsu.

##### Notes

New record: China: Jiangsu (Xinghua), Jiangsu (Zhenze).

#### Reptalus iguchii

(Matsumura, 1914)

2717FFE0-6475-5B44-9B4F-4AFDFC4B9B7F

Oliarus iguchii Matsumura, 1914: 419.| *Reptalusiguchii* (Matsumura, 1914), Rahman, 2011: 35.| Hayashi & Fujinuma, 2016: 326.

##### Distribution

China: Guizhou, Hunan; South Korea: Gyeongsangubuk-do (Rahmam et al. 2011); Japan: Honshu, Kyushu (Hayashi and Fujinuma 2016).

##### Notes

New record: China: Guizhou (Duyun), Guizhou (Tongren), Guizhou (Kaili), Hunan (Suining).

#### Reptalus quadricinctus

(Matsumura, 1914)

9C995D48-7FCE-51A7-874D-9D86083F13DB

Oliarus quadricinctus Matsumura, 1914: 419.| Chou, 1985: 20.| *Reptalusquadricinctus* (Matsumura, 1914), Emeljanov, 1971: 622.| Anufriev & Emeljanov, 1988: 464.| *Reptalusquadricinctus* (Matsumura, 1914), Van Stalle, 1991: 17.| Liang, 2005b 429| Guo & Wang, 2007: 27.| Rahman, 2011: 35.| Bai et al., 2015: 35.| Emeljanov, 2015: 215.| Hayashi & Fujinuma, 2016: 326.

##### Distribution

China: Beijing, Anhui, Fujian, Hunan, Hubei, Jinlin, Shaanxi, Zhejiang, Jiangsu, Shanghai, Sichuan, Guizhou; Japan: Honshu, Kyushu, Shikoku (Hayashi and Fujinuma 2016); Russia: Primorye; South Korea: Daegu (Rahmam et al. 2011).

##### Notes

New record: China: Anhui (Anhui labor university), Shaanxi (Foping nature reserve, Taibai Mountain, Ningqiang, Shiquan, Suining, Chenxi), Hunan (Hupengshan nature reserve, Zhangjiajie nature reserve), Hubei (Houhe nature reserve, Shennongjia), Jilin (Linjiang), Fujian (Shaowu, Huangkeng, Jianning, Daan), Anhui (Anhui labor university), Zhejiang (Fengyangshan), Beijing (Mentougou), Zhejiang (Hangzhou), Jiangsu (Zhenze, Suzhou), Shanghai (Qingpu, Bao Mountain, Sheshan, Jinshan), Sichuan (Qianjiang).

#### Reptalus quinquecostatus

(Dufour, 1833)

C8C3CC1B-1C5B-5AA1-B3C8-1264EA18C1BD

Cixius quinquecostatus Dufour, 1833: 224.| *Reptalusquinquecostatus* (Dufour, 1833), Emeljanov, 1971: 622.| Lodos & Kalkandelen, 1980: 23.| Jovic, 2009: 1055.| Bertin, 2010: 552.| Cvrkovic, 2010: 222.| Jovic, 2010: 238.| Drobnjaković, 2010: 313.| Cvrkovic, 2011: S130.| Mozaffarian & Wilson, 2011: 14.| Emeljanov, 2015: 209.| Mozaffarian, 2018: 480.

##### Distribution

China: Chongqing; Armenia; Austria; Bulgaria; Czech Republic; France; Germany; Greece; Hungary: Andornaktalya; Iran: North (Mozaffarian and Wilson 2011); Italy: Emilia Romagna, Piemonte; Kazakhstan; Portugal; Romania: Csı ´kszereda, Fundulea; Russia: Azov; Serbia: Vršac, Topla, Rajac, South Banat District; Slovakia; Spain; Tadzhikistan; Turkey; Ukraine; Yugoslavia.

##### Notes

New record: China: Chongqing.

#### Reptalus shunxiwuensis

Bai, Guo & Feng, 2015

2C54E1A3-8C56-569D-9ECF-3064DF619F1C

Reptalus shunxiwuensis Bai, Guo & Feng, 2015: 38.

##### Distribution

China: Anhui, Sichuan, Zhejiang (Bai et al. 2015).

#### Siniarus

Emeljanov, 2007

7C738C72-1D63-576A-A240-37DCCE0A647D

#### Siniarus formosanus

(Matsumura, 1914)

A0B4BD3E-6724-538C-BE0E-DE4821EFB11C

Oliarus formosanus Matsumura, 1914: 427.| Van Stalle, 1991: 31.| Schumacher, 1915: 131.| *Siniarusformosanus* (Matsumura, 1914), Emeljanov, 2007: 291.

##### Distribution

China: Taiwan (Matsumura 1914).

#### Siniarus insetosus

(Jacobi, 1944)

0DDF7FC0-CB0E-5549-8000-870C3C256372

Oliarus insetosus Jacobi, 1944: 13.| Fennah, 1956: 454.| *Siniarusinsetosus* (Jacobi, 1944), Emeljanov, 2007: 291.

##### Distribution

China: Fujian (Jacobi 1944), Guangdong, Hongkong, Hubei, Sichuan (Fennah 1956), Yunnan, Tibet, Guangxi, Guizhou, Hunan, Taiwan.

#### Siniarus scalenus

(Tsaur, Hsu & Van Stalle, 1988)

9383C656-69FC-50C4-AE0C-56A55E9310B3

Oliarus scalenus Tsaur, Hsu & Van Stalle, 1988: 41.| Van Stalle, 1988: 29.| *Siniarusscalenus* (Tsaur, Hsu & Van Stalle, 1988), Emeljanov, 2007: 291.

##### Distribution

China: Taiwan (Tsaur et al. 1988).

#### Semonini

Emeljanov, 2002

FBFC17DE-073E-55D6-8618-E563C0552F16

#### Betacixius

Matsumura, 1914

B562C45F-0AC5-505A-9DD5-FEC7D0AD617A

#### Betacixius bispinus

Zhang & Chen, 2011

3ECEAD8C-6927-5C0D-87E5-2E2B5176C8BD

Betacixius bispinus Zhang & Chen, 2011b: 53.

##### Distribution

China: Sichuan (Zhi et al. 2020a), Guangxi, Guizhou (Zhang and Chen 2011b), Xinjiang, Yunnan (Zhi et al. 2020a).

##### Notes

New record: China: Xinjiang (Changji Temple), Guangxi (Lintian).

#### Betacixius brunneus

Matsumura, 1914

371E1BC6-F487-5B61-AC3B-AAE7FD621361

Betacixius brunneus Matsumura, 1914: 417.| Hori, 1982: 181.| Tsaur et al., 1991b: 37.| Zhang & Chen, 2011b: 50.| Hayashi & Fujinuma, 2016: 323.

##### Distribution

China: Fujian, Zhejiang, Taiwan (Zhang and Chen 2011b); Japan; Nansei-shoto: Ryukyu Islands (Hayashi and Fujinuma 2016).

##### Notes

New record: China: Fujian (Taoyuan valley scenic spot of wuyi Mountain).

#### Betacixius clypealis

Matsumura, 1914

EC017CC6-4405-5320-BC10-7634F0C17C7D

Betacixius clypealis Matsumura, 1914: 415.| Hori, 1982: 181.| Tsaur et al., 1991b: 39.

##### Distribution

China: Zhejiang, Taiwan (Matsumura 1914).

##### Notes

New record: China: Zhejiang (Jiulong Mountain, Wuyanling).

#### Betacixius clypealis vitifrons

(Matsumura, 1914)

7A97A4B6-E069-5308-843C-3CAF9ADB3C5D

Betacixius clypealis vitifrons Matsumura, 1914: 416.

##### Distribution

China: Taiwan (Matsumura 1914).

#### Betacixius delicates

Tsaur & Hsu, 1991

B6D64390-B163-5DCF-B070-0ECFF462B375

Betaxixius delicates Tsaur & Hsu in Tsaur et al., 1991a: 29.

##### Distribution

China: Shaanxi, Zhejiang, Yunnan, Taiwan (Tsaur et al. 1991a).

##### Notes

New record: China: Zhejiang (Fengyang Mountain).

#### Betacixius euterpe

Fennah, 1956

387E6F11-676E-5101-B53B-A7246A3EF440

Betaxixius euterpe Fennah, 1956: 458; Zhang & Chen, 2011b: 50.

##### Distribution

China: Guangdong (Fennah 1956).

#### Betacixius flagellihamus

Zhang & Chen, 2011

B0735B12-8826-5BB9-AC0D-C1FA08E668ED

Betacixius flagellihamus Zhang & Chen, 2011b: 54.

##### Distribution

China: Guizhou (Zhang and Chen 2011b).

#### Betacixius flavovittatus

Hori, 1982

69889B0C-1100-54D2-9CD0-8656D17437F1

Betacixius flavovittatus Hori, 1982: 179.| Tsaur et al., 1991a: 41.| Zhang & Chen, 2011b: 50.

##### Distribution

China: Zhejiang, Taiwan (Tsaur et al. 1991a).

##### Notes

New record: China: Zhejiang (Fengyang).

#### Betacixius fuscus

Tsaur & Hsu, 1991

7B432300-AA91-5F22-8411-20CFB1D98202

Betacixius fuscus Tsaur & Hsu in Tsaur et al., 1991a: 44.| Zhang & Chen, 2011b: 50.

##### Distribution

China: Fujian, Taiwan (Tsaur et al. 1991a).

##### Notes

New record: China: Fujian (Longyan City Contour Park).

#### Betacixius herbaceous

Tsaur & Hsu, 1991

B11F5E96-C220-58F4-A9B7-91BFF799A547

Betacixius herbaceous Tsaur & Hsu in Tsaur et al., 1991a: 28.

##### Distribution

China: Yunnan, Taiwan (Tsaur et al. 1991a).

##### Notes

New record: China: Yunnan (Yangyang Valley, Matang Reservoir).

#### Betacixius latistilus

Zhi, Zhang, Yang & Chen, 2020

6FA31B19-DE44-5A14-945E-F7A1591121EF

Betacixius latistilus Zhi, Zhang, Yang & Chen, 2020a: 8.

##### Distribution

China: Yunnan (Zhi et al. 2020a).

#### Betacixius maculosus

Tsaur & Hsu, 1991

BC2FDE65-2DC5-5F98-80A3-F6A484D75011

Betacixius maculosus Tsaur & Hsu in Tsaur et al., 1991a: 31.

##### Distribution

China: Fujian, Sichuan, Taiwan (Tsaur et al. 1991a).

##### Notes

New record: China: Fujian (Wuyi Mountain), Sichuan (Emei Mountain).

#### Betacixius maguanensis

Zhi, Zhang, Yang & Chen, 2020

36145D78-AD41-5AB9-A980-89D2D1360AD6

Betacixius maguanensis Zhi, Zhang, Yang & Chen, 2020a: 11.

##### Distribution

China: Yunnan (Zhi et al. 2020a).

#### Betacixius michioi

Hori, 1982

33F280E6-1E48-510F-8254-EEA650569DE8

Betacixius michioi Hori, 1982: 176.| Tsaur et al., 1991a: 35.| Zhang & Chen, 2011b: 50.

##### Distribution

China: Yunnan, Taiwan (Tsaur et al. 1991a).

##### Notes

New record: China: Yunnan (Yangyang Valley, Matang Reservoir).

#### Betacixius nelides atrior

Fennah, 1956

DB1C8EDB-3BCA-58A9-B5BD-96327C975828

Betacixius nelides atrior Fennah, 1956: 458.

##### Distribution

China: Zhejiang (Fennah 1956).

#### Betacixius nelides nelides

Fennah, 1956

F6669645-CBC6-590D-8098-25E563C46C0B

Betacixius nelides nelides Fennah, 1956: 457.

##### Distribution

China: Guangdong (Fennah 1956).

#### Betacixius nigromarginalis

Fennah, 1956

F62DA16A-AFA8-53BF-858B-8024AD3B8B1C

Betacixius nigromarginalis Fennah, 1956: 457.

##### Distribution

China: Hubei (Fennah 1956).

#### Betacixius obliquus

Matsumura,1914

BBD47E53-5BB4-554F-B004-1FF59C75C034

Betacixius obliquus Matsumura, 1914: 414.| Chou, 1985: 23; Chou, 1998: 382.| Liang, 2005b: 429.| Zhang & Chen, 2011b: 50.| *Betacixiusobliquus* (Matsumura, 1914), Hayashi & Fujinuma, 2016: 323.

##### Distribution

China: Fujjian, Guizhou, Guangxi, Guangdong, Hainan, Hunan, Sichuan (Liang 2005b), Yunnan, Zhejiang; Japan: Honshu, Kyushu, Shikoku (Hayashi and Fujinuma 2016).

##### Notes

New record: China: Hainan (Diaoluoshan, Limu Island), Fujian (Wuyi Mountain, Chongan, Guangze, Yongan), Sichuan (Emei Mountain), Guangxi (Huaping nature reserve), Hunan (Shenzhou); Guangdong (Dinghu Mountain), Zhejiang (Qingyuan, Longquan).

#### Betacixius ocellatus

Matsumura, 1914

BA74F6E8-0C47-55E1-B58D-22507F108C95

Betacixius ocellatus Matsumura, 1914: 412.| Esaki 1932: 1774.| Hori, 1982: 181.| Tsaur et al., 1991b: 33.

##### Distribution

China: Yunnan, Fujian, Taiwan (Tsaur et al. 1991a).

##### Notes

New record: China: Fujian (Shaowu Jiangshi Nature Reserve).

#### Betacixius pallidior

Jacobi, 1944

F74FBC5A-7A57-5448-82F5-15BBB71C094C

Betacixius pallidior Jacobi, 1944: 15.| Fennah, 1978: 213.

##### Distribution

China: Fujian (Jacobi 1944); Vietnam: Hanio (Fennah 1978).

#### Betacixius rinkihonis

Matsumura, 1914

FD96DF70-28BA-5890-A052-633254C51AA7

Betacixius rinkihonis Matsumura, 1914: 417.| *Betacixiusrinkihonis* Hori, 1982: 180.| Tsaur et al., 1991a: 42.

##### Distribution

China: Guangdong, Taiwan (Tsaur et al. 1991a).

##### Notes

New record: China: Guangdong (Shaoguan Nanling).

#### Betacixius robustus

Jacobi, 1944

FB8B66DF-2902-5884-AEB8-6F0BE4235395

Betacixius robustus Jacobi, 1944: 15.| Zhang & Chen, 2011b: 50.

##### Distribution

China: Fujian (Jacobi 1944).

#### Betacixius shirozui

Hori, 1982

8F93EDED-0633-51FF-AC3F-F225587812C0

Betacixius shirozui Hori, 1982: 178.| Tsaur et al., 1991a: 48.| Zhang & Chen, 2011b: 50.

##### Distribution

China: Yunnan, Taiwan (Tsaur et al. 1991a).

##### Notes

New record: China: Yunnan (Mengla).

#### Betacixius sparsus

Tsaur & Hsu, 1991

884E2AE0-57E2-5A42-AAE9-B4A4120AEF6E

Betacixius sparsus Tsaur & Hsu in Tsaur et al., 1991a: 46.| Zhang & Chen, 2011b: 50.

##### Distribution

China: Fujian, Hainan, Taiwan (Tsaur et al. 1991a).

##### Notes

New record: China: Fujian (Longyan), Hainan (Jianfengling).

#### Betacixius transversus

Jacobi, 1944

583020E7-D2B4-5AB0-9BE3-08F8347C9AAF

Betacixius transversus Jacobi, 1944: 14| Zhang & Chen, 2011b: 50.

##### Distribution

China: Fujian (Jacobi 1944).

#### Kuvera

Distant, 1906

FB87F526-3FC0-5A82-B43F-DCC310C70806

#### Kuvera communis

Tsaur & Hsu, 1991

65535261-09A3-56A8-9C5B-BDD54C5EEF38

Kuvera communis Tsaur & Hsu in Tsaur et al., 1991a: 59.

##### Distribution

China: Fujian, Taiwan (Tsaur et al. 1991a).

##### Notes

New record: China: Fujian (Fengyang Mountain).

#### Kuvera flaviceps

(Matsumura, 1900)

129E9CF7-62D5-5ABA-BB45-23871731E7D1

Oliarus flaviceps Matsumura, 1900: 208.| *Kuveraflaviceps* (Matsumura, 1900), Matsumura, 1914: 407.| Anufriev, 1987: 14.| Anufriev & Emeljanov, 1988: 449.| Hayashi & Fujinuma, 2016: 325.

##### Distribution

China: Gansu, Jilin; Japan: Hokkaido, Honshu, Kyushu, Shikoku (Hayashi and Fujinuma 2016); Korea; Russia: Sakhalin.

##### Notes

New record: China: Gansu (Wen County).

#### Kuvera hama

Tsaur & Hsu, 1991

9C958D3C-7652-57F4-8D56-BB710E37238A

Kuvera hama Tsaur & Hsu in Tsaur et al., 1991b: 61.

##### Distribution

China: Jilin, Fujian, Hunan, Taiwan (Tsaur et al. 1991a).

##### Notes

New record: China: Fujian (Wuyi Mountain), Hunan (Huping Mountain).

#### Kuvera huoditangensis

Luo, Liu & Feng, 2019

77B700A1-797F-581D-B651-806F8D1B5297

Kuvera huoditangensis Luo, Liu & Feng, 2019a: 140.

##### Distribution

China: Shaanxi (Luo et al. 2019a), Henan, Gansu.

#### Kuvera kurilensis

Anufriev, 1987

4E89264D-560C-5DE2-8899-690247C9AA5E

Kuvera kurilensis Anufriev, 1987: 15.| Anufriev & Emeljanov, 1988: 449.

##### Distribution

China: Jilin, Fujian, Hunan, Tibet, Taiwan; Russia: Kuril Islands (Anufriev 1987).

##### Notes

New record: China: Tibet (Motlin Green).

#### Kuvera laticeps

(Metcalf, 1936)

6A6A657F-5A45-537B-AD4C-4DD814A2C982

Cixius latifrons Melichar, 1902: 85.| *Cixiuslaticeps* Metcalf, 1936: 180.| *Kuveralaticeps* (Metcalf, 1936), Anufriev, 1987: 6.

##### Distribution

China: Sichuan (Anufriev 1987).

#### Kuvera longipennis

Matsumura, 1914

C4A02E79-A942-5714-A059-FF2FCE4F1BE4

Kuvera longipennis Matsumura, 1914: 411.

##### Distribution

China: Taiwan (Matsumura 1914).

#### Kuvera longwangshanensis

Luo, Liu & Feng, 2019

EAE4AA75-930F-5AE3-B29D-753E8A91C00A

Kuvera longwangshanensis Luo, Liu & Feng, 2019a: 144.

##### Distribution

China: Zhejiang (Luo et al. 2019a).

#### Kuvera pallidula

Matsumura, 1914

1BB998C4-309B-5675-A196-2FDF0E36E6B9

Kuvera flaviceps var. pallidula Matsumura, 1914: 409.| *Kuverapallidula* Matsumura, 1914, Anufriev, 1987: 10.| Anufriev & Emeljanov, 1988: 449.| Hayashi & Fujinuma, 2016: 325.

##### Distribution

China: Jilin, Guangxi, Shaanxi, Sichuan; Japan: Hokkaido, Honshu (Hayashi and Fujinuma 2016); Russia: Far East (Anufriev 1987).

##### Notes

New record: China: Jilin (Antu County), Guangxi (Longsheng), Sichuan (Yaan).

#### Kuvera semihyalina

Distant, 1906

96AA91AF-3556-5F60-8835-FBEEF4AEF377

Kuvera semihyalina Distant, 1906: 261.| Anufriev, 1987: 6.

##### Distribution

China: Liaoning, Shaanxi; Myanmar: (Distant 1906).

##### Notes

New record: China: Liaoning (Baling County National Balding National Nature Reserve), Shaanxi (Shiquan, Qinling).

#### Kuvera similis

Tsaur & Hsu, 1991

F45EE93D-1970-5AB4-843B-E3D32669EACC

Kuvera similis Tsaur & Hsu in Tsaur et al., 1991a: 55.

##### Distribution

China: Beijing, Fujian, Taiwan (Tsaur et al. 1991a).

##### Notes

New record: China: Beijing (Mentougou), Fujian (Meihua).

#### Kuvera taiwana

Tsaur & Hsu, 1991

6EEDB20E-7200-5845-8AEF-7DB1C47D2D5B

Kuvera taiwana Tsaur & Hsu in Tsaur et al., 1991a: 50.

##### Distribution

China: Hainan, Shaanxi, Yunnan, Ningxia, Zhejiang, Tibet, Taiwan (Tsaur et al. 1991a).

##### Notes

New record: China: Zhejiang (Fengyang Mountain, Linan), Hainan (Yinggeling), Tibet (Yadong); Shaanxi (Huayin), Yunnan (Lvchun), Ningxia (Liupan Mountain).

#### Kuvera tappanella

Matsumura, 1914

91C92FDC-C07B-5578-BF0A-AD874DABE762

Kuvera tappanella Matsumura, 1914: 410.

##### Distribution

China: Hubei, Jiangxi, Hunan, Jilin, Taiwan (Tsaur et al. 1991a).

##### Notes

New record: China: Hubei (Shennongjia), Jiangxi (Jinggang Mountain), Hunan (Mang Mountain), Hubei (Shennongjia), Jilin (Changbai Mountain).

#### Kuvera toroensis

Matsumura, 1914

FF492551-9878-5301-8E1C-6CA89A5E770E

Kuvera toroensis Matsumura, 1914: 410.| Anufriev, 1987: 18.

##### Distribution

China: Yunnan, Jiangxi, Zhejiang, Hunan, Tibet, Taiwan (Matsumura 1914).

##### Notes

New record: China: Hunan (Qianyang), Jiangxi (Lu Mountain), Yunnan (Mengla Longmen, Matang Reservoir), Zhejiang (Hangzhou), Tibet (Langxian Cuona).

#### Kuvera transversa

Tsaur & Hsu, 1991

251304F6-F611-5954-AC49-EF09A0D16160

Kuvera transversa Tsaur & Hsu in Tsaur et al., 1991a: 57.

##### Distribution

China: Yunnan, Taiwan (Tsaur et al. 1991a).

##### Notes

New record: China: Yunnan (Tengchong Laifeng Mountain).

#### Kuvera ussuriensis

(Vilbaste, 1968)

3602C65F-A0E0-58BA-80DF-D9921DEC7E14

Betacixius ussuriensis Vilbaste, 1968: 9.| *Kuveraussuriensis* (Vilbaste, 1968), Anufriev, 1987: 17.

##### Distribution

China: Sichuan; Russia: Primorsky Territory, South of the Khabarovsk Territory (Anufriev 1987); Japan: Hokkaido.

#### Kuvera vilbastei

Anufriev, 1987

FFA1C0F2-3824-5C85-A022-9DD879306BB7

Kuvera vilbastei Anufriev, 1987: 7.| Anufriev & Emeljanov, 1988: 448.| Anufriev, 2009: 68.

##### Distribution

China: Shaanxi, Zhejiang, Tibet; Russia: Primorye (Anufriev 1987).

##### Notes

New record: China: Tibet (Bomi Yigong, Yadong), Shaanxi (Hua Mountain, Huxian), Zhejiang (Tianmu Mountain).

#### Kuvera yecheonensis

Rahman, Kwon & Suh, 2017

CA31306D-26BD-5B62-BFA3-2B84C19DB6A4

Kuvera yecheonensis Rahman, Kwon & Suh, 2017: 10.

##### Distribution

China: Guizhou; South Korea: Gyeongsangbuk-do (Rahman et al. 2017).

##### Notes

New record: China: Guizhou (Qiandong).

#### Stenophlepsiini

Metcalf, 1938

3FEB49C8-1220-5935-B0E4-2B3DBE3A1E45

#### Euryphlepsia

Muir, 1922

0CB5516F-E7C6-566A-8782-904DD3844A0A

#### Euryphlepsia yamia

Tsaur, 1989

2E9092F0-220C-584F-8FE8-25C805F4E67D

Euruphlepsia yamia Tsaur, 1989: 82.

##### Distribution

China: Taiwan (Tsaur 1989b).

## Analysis

### Checklist

Ten cixiid tribes are reported in China: Cixiini Spinola, 1839, Oecleini Muir, 1922, Bennini Metcalf, 1938, Stenophlepsiini Metcalf, 1938, Pentastirini Emeljanov, 1971, Borysthenini Emeljanov, 1989, Emeljanov, 2002; Brixiini Emeljanov, 2002, Eucarpiini Emeljanov, 2002, and Semonini Emeljanov, 2002. These tribes include 35 genera and subgenera, 250 species and 400 collection records from 28 Chinese provinces. In this study, 77 new species were recorded for the first time from China.

### Regional richness and endemism

A species richness gradient occurs from north to south and from west to east for Cixiidae as shown in Fig. 2. Substantial variation in species richness and endemism among the different zoogeographic regions were observed. Table 1 describes the species richness of Cixiidae by region, ranging from 5 species and no endemic species in the Nei Mongol-Xinjiang region, to 161 species and 69.57% of endemic species in Taiwan region. In-between, species richness and endemism ratios are distributed in two groups: the Northeast China and the Qinghai-Tibet regions, respectively with 8 and 10 species and 12.5% and 20% of endemism, and the North, Southwest and Central China regions, which have comparable numbers of species and endemism, respectively, with 29, 43 and 60 species and 33-40% of endemism. No significant differences in endemism among regions was observed. More than five-fourths of the species (205 species; 82%) are reported to occur in only in China, depicting a high level of endemism of the Chinese fauna for this family (Table 1).

### Distribution patterns of cixiid species in China

Based on the eight zoogeographic regions of China (Fig. 1), 38 main distribution patterns are observed (Table 2). The number of species distributed in a single region (accounting for regional endemism) is highly variable among the regions: Taiwan (44.80%), Central China (8.00%), Southwest China (6.80%), South China (5.60%), North China (4.00%), Qinghai-Tibet (0.80%) and Northeast China (0.40%). No endemic species were observed in the Nei Mongol-Xinjiang region (Table 2). Nine bi-regional distribution patterns were observed, and among them the South China-Taiwan pattern has the greatest number of species (15 species, 6.00% of the species). Nine tri-regional distribution patterns were also observed, among which, the largest number of species (11 species, 4.40% of the species) was for the Central-South China-Taiwan distribution pattern. The Southwest-South China-Taiwan distribution pattern is depicted by 6 species (2.40% of the species). Five distribution patterns occur in 4 zoogeographic regions, among which the North Southwest-Central-South China region and the Northeast-Central-South China-Taiwan region have two species (0.80% of the total number of cixiids in China). All the remaining four-, five-, six- and seven-regional distribution patterns have only a single species, accounting for 0.40% of the total number of cixiids in China (Table 2).

### Cixiid patterns of distribution at the tribal level and generic level

**Figure 4.**
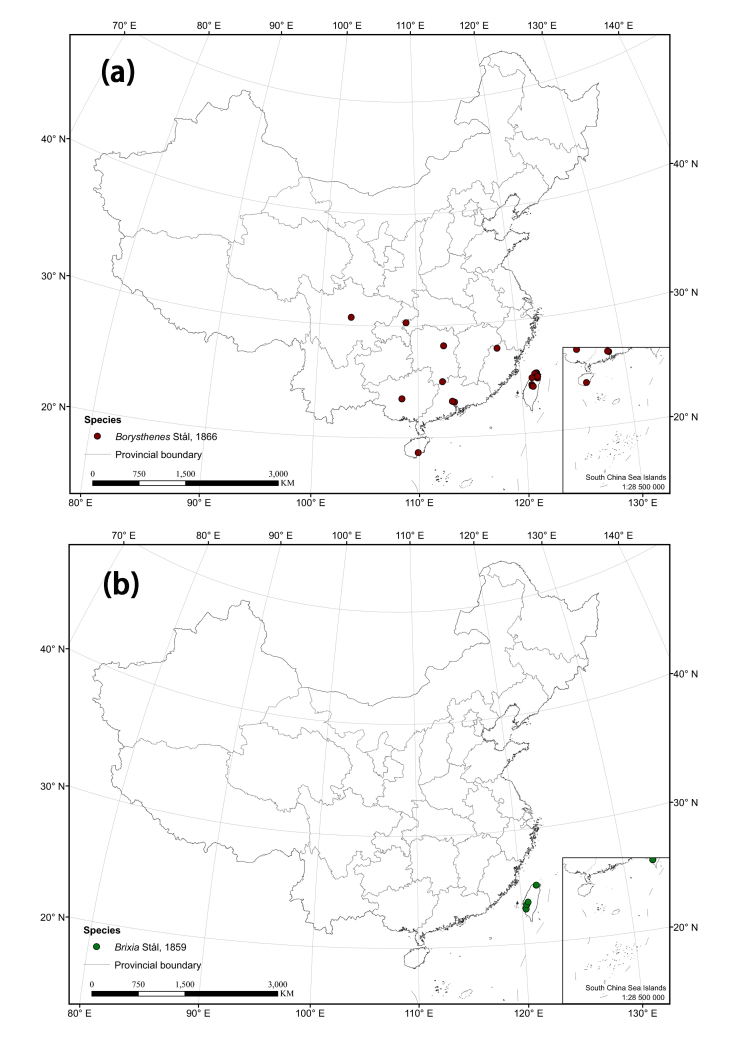
Distribution of records of the tribes Borysthenini and Brixiini in China. (a) Borysthenini Emeljanov, 1989; (b) Brixiini Emeljanov, 2002.

**Figure 5.**
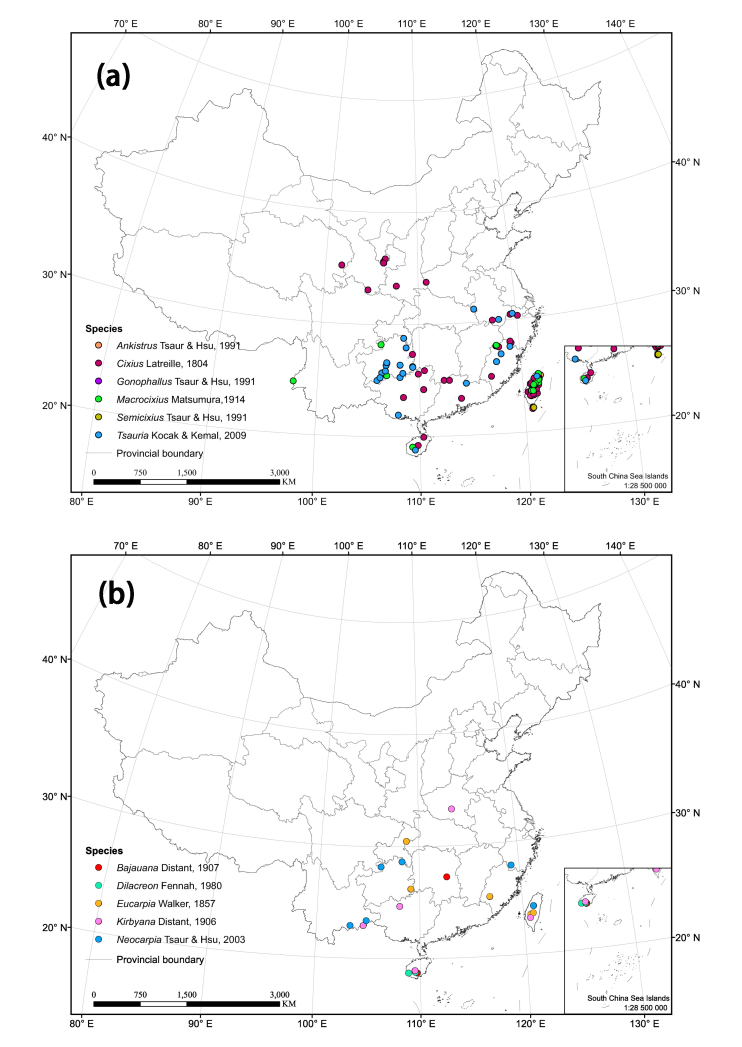
Distribution of records of the tribes Cixiini and Eucarpiini in China. (a) Cixiini Spinola, 1839; (b) Eucarpiini Emeljanov, 2002.

**Figure 6.**
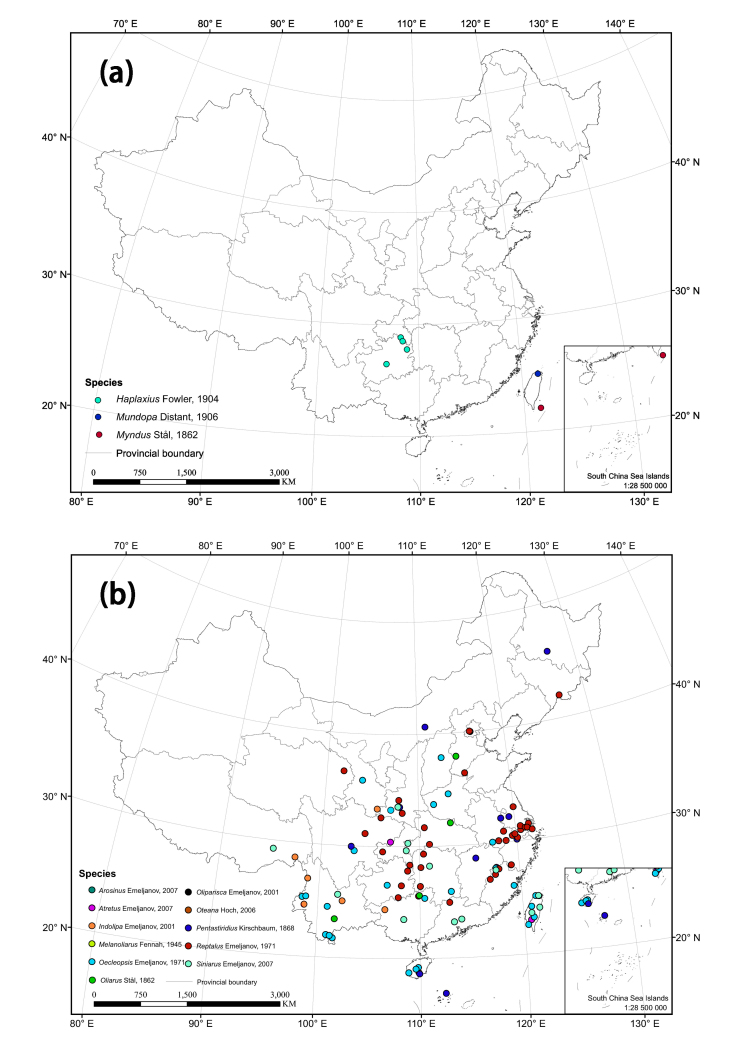
Distribution of the tribes Oecleini and Pentastirini in China. (a) Oecleini Muir, 1922; (b) Pentastirini Emeljanov, 1971.

**Figure 7.**
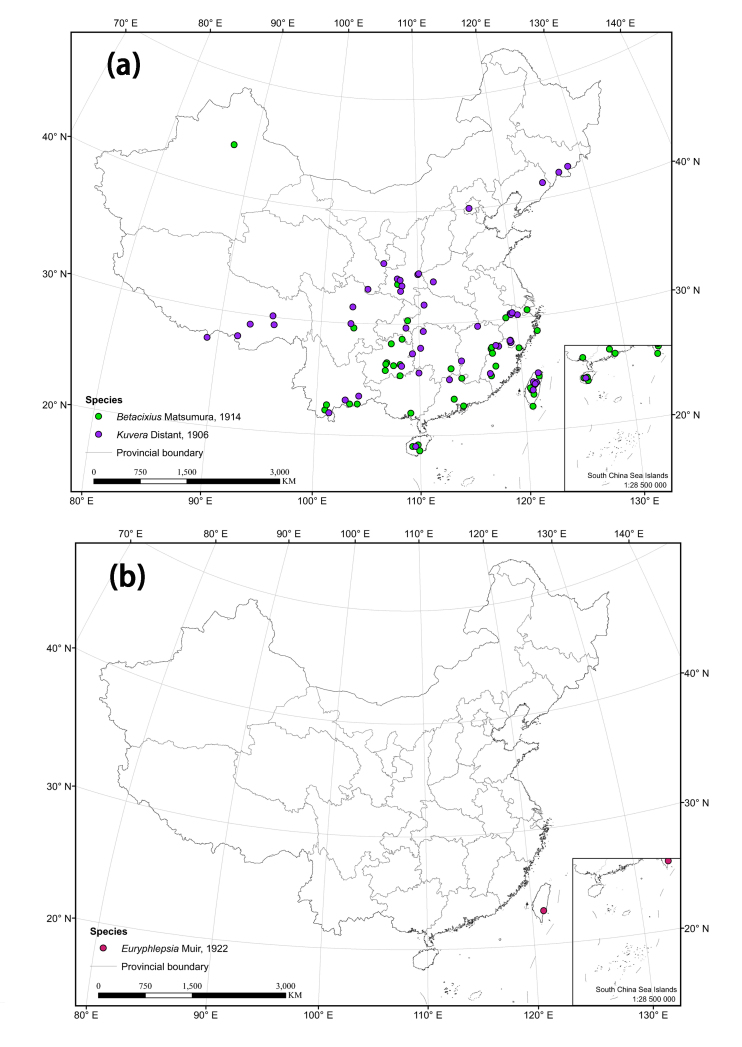
Distribution of the tribes Semonini and Stenophlepsiini in China. (a) Semonini Emeljanov, 2002; (b) Stenophlepsiini Metcalf, 1938.

Of the ten cixiid tribes distributed in China (Table 3), Pentastirini (21.20%, Fig. 6b) and Semonini (17.20%, Fig. 7a) are the two most widely distributed tribes in China. Cixiini (Fig. 5a), which is the most species-rich tribe with 45.20% of the species, is distributed in 7 regions of China, but has not been reported from the Northeast China region. Andini (5.20%, Fig. 3a) is not distributed in the Palaearctic realm in China; Eucarpiini (6.40%, Fig. 5b) and Borysthenini (2.00%, Fig. 4a) are distributed only in the Southwest, Central, South China, and in the Taiwan regions. The remaining tribes, Bennini (0.40%, Fig. 3b), Brixiini (0.80%, Fig. 4b), Oecleini (1.20%, Fig. 6a) and Stenophlepsiini (0.40%, Fig. 7b) are only found in Taiwan.

Thirty-three Cixiidae genera are present in China (Table 4; Fig. 3; Fig. 4; Fig. 5; Fig. 6; Fig. 7), with *Kuvera* (18 species, 7.2%) being the most widespread genus in China although *Pentastiridius* (5 species, 2%) is only unreported from the Qinghai-Tibet region. *Cixius* is the most diverse (95 species, 38%) but is not distributed in the Southwest and Northeast China regions. *Betacixius* (25 species, 10%) is not distributed in the Northeast China and Qinghai-Tibet regions. *Oliarus* and *Reptalus* (each with 6 species 2.4%) are both undistributed in the Nei Mongol-Xinjiang region, while the former is not reported from the Qinghai-Tibet region and the latter not reported from the Taiwan region. *Oliparisca* (1 species, 0.4%) is only distributed in the Qinghai-Tibet region and 10 genera are only reported from the Taiwan region. In addition, we also found that 16 genera are all distributed in the south of Sino-Japanese/Oriental boundary.

### Cluster and Ordination

**Figure 8.**
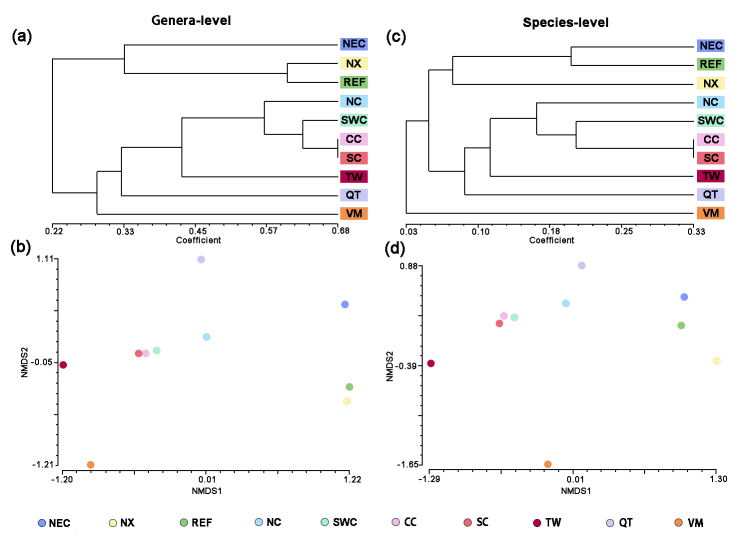
Dendrograms from UPGMA clustering and NMDS ordination of Jaccard similarity coefficients based on Chinese zoogeographical regions and adjacent areas for Chinese Cixiidae genera (a), (b) and species (c), (d). Abbreviations: NEC, Northeast China; NC, North China; NX, Nei Mongol-Xinjiang; QT, Qinghai-Tibet; SWC, Southwest China; CC, Central China; SC, South China; TW, Taiwan; RFE, Russian Far East; VM, Vietnam, Laos, Thailand, Cambodia, Myanmar, Bhutan, Bangladesh and part of Indian.

In both the generic and specific taxonomic levels (Fig. 8a, c), the dendograms clearly separate the northernmost regions (Russian Far East, Nei Mongol-Xinjiang and Northeast China regions) from all other regionsand with the similar relationships for Chinese zoogeographical regions: South-Central + SouthWest + North + Taiwan + Qinghai-Tibet. At the species-level the south adjacent China country region appears as sister to all of China. In contrast, at the generic level, this south adjacent China region sister to the central and south Chinese regions. In the northernmost regions, Russian Far East is closer to the Northeast China region at the species level and closer to the Nei Mongol-Xinjiang region at the generic level. In both analyses, the cophenetic correlation coefficient (r>0.8) is high, indicating close agreement between the cluster assignment and the original Jaccard similarity coefficient matrix.

The cluster analysis and the NMDS ordination generally showed similar interrelationships among regions (Fig. 8b, d). The stress values of 0.18 (generic level) and 0.30 (species level) demonstrate the accuracy of the projections in the matrix in the 2D ordination space. At the generic level (Fig. 8b), the Nei Mongol-Xinjiang and Russian Far East regions are closely related to each other, and the Northeast China, Nei Mongol-Xinjiang, and Russian Far East regions are clearly separated from the other 7 regions. The Southwest, Central, and South China regions are closely grouped together, and are also related to the North China and Taiwan regions, but the Qinghai-Tibet and VM regions are more separated. At the species level (Fig. 8d), a roughly similar pattern occurs and the Russian Far East is closer to the Northeast China region, but the VM region is clearly separated and more distant from all other regions.

## Discussion

### Current Chinese Cixiidae diversity and distribution

More than 80% of the Cixiidae species are considered to be endemic to China. The highest endemism is found in Taiwan (69.57%), followed by the Southwest China (39.53%), North China (34.48%) and Central China (33.33%) regions. These figures are consistent with the species richness and endemism patterns observed in other Hemiptera groups, such as aphids (Huang et al. 2008, Gao et al. 2018), leafhoppers (Yuan et al. 2014), or more specifically for planthoppers (Zhao et al. 2020a, Zhao et al. 2020b). For the patterns of distribution, the South China-Taiwan pattern (6.00%), the Central-South China-Taiwan one (4.40%) and the South-Western-South China-Taiwan one (2.40%) are the richer in term of species. This pattern probably results from the past interconnection of the island of Taiwan with the Asian continent during the Quaternary period, when the sea level fell, facilitating the species flow between these areas (Lei et al. 2003, Tang et al. 2006). Its subsequent geographical isolation after the Quaternary period explains its relatively independent pattern of speciation (Gao et al. 2018) and its high endemicity of species.

At the tribal level Cixiini, Pentastirini, and Semonini are widely distributed in China, except i the Northeastern China region for the Cixiini, which is probably a collect artefact as Cixiini are known to occur in higher latitudes (Bourgoin 2021). With 5.20% of the species, Andini is distributed in the Sino-Japanese - Oriental Region (NC, QT, SWC, CC, SC, TW), and Eucarpini and Borysthenini (6.40% and 2.00% respectively) are mainly concentrated south to the Qingling Mountain-Huai River (SWC, CC, SC, TW). The remaining four tribes [Bennini (0.40%), Briixini (0.80%), Oecleini (1.20%) and Stenophlepsiini (0.40%)] are all distributed in Taiwan.

At the generic level, *Kuvera* (7.2%) is the most widely distributed genus in China. *Pentastiridius* (2%) is not distributed in the Qinghai-Tibet region. *Cixius* (38%) is not distributed in the Southwest and Northeast China regions, but the genus was reported from the Russian Far East, so it may be a collection bias. In addition, one genus is distributed only in the Tibet region, while 10 genera are distributed only in the Taiwan region. We also found that nearly half of the genera (16 genera, 48.48%) are distributed south of the Sino-Japanese/Oriental boundary.

### Biogeographical history shapped Chinese Cixiidae diversity

Cixiidae have a wide range of host plants, including mostly angiosperm Eudicot shrubs: Asterales, Rosales, Fabales, Myrtales, Lamiales, etc., but also Monocots as Poales and Arecales and tall trees such as Gymnosperm Pinales, or Magnolids Laurales, etc (Bourgoin 2021). Known from fossil records since the Barremian period, 130MYA (Luo et al. 2020) and probably occurring at least since 200MYA (Urban and Cryan 2012, Song and Liang 2013, Johnson et al. 2018), it is likely that the radiation of angiosperms around 145 MY (Condamine et al. 2020), has greatly influenced the diversification of the major Cixiidae lineages (Labandeira 2014, Szwedo 2016, Luo et al. 2020).

More recently, the uplift of the Qinghai-Tibetan Plateau starting in the middle of the Eocene period (45-38 Ma), also had profound effects on the topography and watersheds of East Asia, the aridity of inland Asia, and the Asian monsoon system. These abiotic factors produced a three-stage pattern of species distribution, from high in the west to low in the east (Zhang et al. 2000). The vertical differentiation in plant distribution (Jin et al. 2003), affected their diversity and inceased the richness of local speciation events (Wen et al. 2014, Favre et al. 2015, Ye et al. 2017), and subsequently influenced the species distribution and speciation of the Cixiidae. During the late Oligocene to early Miocene periods (25-15Ma), the expansion of the Tibetan Plateau continued, and the East Asian monsoon and Indian monsoon prevailed in the Asian continent. This resulted in an increase of both temperature and sea levels (Ye et al. 2017), which allowed the northward propagation of fauna and flora. This area was pushed back southwards at the end of the Miocene period (10 Ma) by the uplift of the Hengduan Mountains (Li et al. 2020), which caused the climate to cool (Xie et al. 2019, Yu et al. 2020). Since the middle of the Holocene period (6Ka), rainfall declined and monsoon strength weakened, resulting in a dramatic decrease in precipitation in northern China, which affected the vegetative environment (Zhao et al. 2009, Huang et al. 2012). Quantitative precipitation reconstructions based on pollen collected from northern China indicated that a strong sea-land pressure and temperature gradient caused by strong summer insolation in the northern hemisphere during the early Holocene period (0.14-0.07 Ma) caused enhanced monsoons (Zhao et al. 2009, Cook et al. 2011, Chen et al. 2015, Ge et al. 2017). Obviously the cixiid fauna diversity fluctuated at the same periods along with the diversity of their host-plants. However, without more robust phylogeny studies of Cixiidae, it remains difficult to better infer a more precise biogeographic historical scenario for the family and to link their distribution patterns to any of these important past events.

### Biogeographical patterns of Chinese cixiids

Traditionally, the global biogeographical regionalization of China covers both the Oriental and Palearctic realms, which are bounded by the Qingling Mountain-Huai River, around 32–34N in the east of China (Sclater 1858, Wallace 1876, Zhen 1960, Zhang 1999, Cox 2001, Kreft and Jetz 2010, Morrone 2015, Song et al. 2016, He et al. 2017). In 2013, based on its zoological fauna, Holt added a Sino-Japanese realm standing between the Palearctic and Oriental realms, and from west of Tibet to east of the Japanese archipelago. He located the Palaearctic/Sino-Japanese boundary at about 40–41N, and the Sino-Japanese/Oriental boundary at 24–25N in southeast China (Fig. 1). Kreft and Jetz (2013) questioned the validity of this realm because they regarded it as just a biogeographical transition zone between the Palearctic and Oriental realms. According to their taxa etho-ecological characteristics, the Sino-Japanese realm boundaries are generally clustered with the Oriental realm (Kreft and Jetz 2010, Song et al. 2016, He et al. 2017, Gao et al. 2017, He et al. 2018).

This result is also observed here for the Chinese Cixiidae divided into two major zoogeographic areas: the Nei Mongol-Xinjiang and Northeast China regions from the rest of China. This boundary corresponds to the Palearctic/Sino-Japanese north boundary and appears to be more well defined than the Palearctic/Oriental boundary. The Andini tribe serves as a landmark for the Palearctic/Sino-Japanese north boundary, while the Eucarpini and Borysthenini tribes are primarily concentrated south to the Qingling Mountain-Huai River point to the traditional Palaearctic/Oriental boundary as proposed by Zhang (2011). Eucarpini and Borysthenini are landmarks for the south Sino-Japonese realm, clustering with the Oriental realm. Bennini, Briixini, Oecleini and Stenophlepsiini, which are all distributed in Taiwan, may either indicate the northern limit of older and wider distributions of these tribes or might have resulted from occasional dispersions from neighbouring south regions.

At the genus level, the south parts of China cluster with the Indochina region in our analyses, but at the species level all of China forms a unique group. This may be related to the late Eocene uplift of the Himalayas and recent uplift of the Himalayan-Hengduan Mountains in the late Miocene, with a peak before the late Pliocene (Harrison et al. 1992, Sun et al. 2011). These geographical uplifts resulted in the formation of large topographic barriers isolating South China fauna and favorizing recent speciation events and endemism as already shown in several other taxa such as frogs (Che et al. 2010), insects (Ye et al. 2016; Chen et al. 2016), birds (Liu et al. 2016, Cai et al. 2018, Dong et al. 2020), mammals (Ge et al. 2017) and plants (Feng et al. 2013, Favre et al. 2016, Ebersbach et al. 2017, Liu et al. 2017, Ye et al. 2017). Moreover, Quaternary (2.6 Ma) tectonic movements and the influence of the Indian and Pacific monsoons greatly contributed also to the segregation, dispersal and speciation of Cixiidae in southern China and Southeast Asia (Shi et al. 1998, Liang 2003).

The South China region is usually included in the Oriental realm in other studies (Zhang 1999, Zhang 2011), but our analysis indicates that for Cixiidae the South China region is closer to the Central, Southwes, and North China region (Sino-Japonese realm). This is consistent with the results of a quantitative analysis of terrestrial mammals in China and adjacent regions by Xiang et al. (2004), where clustering analysis showed the proximity of South China region to Central and Southwest China regions, and they suggested these regions as the South China Division.

### Conclusions

This study is the first zoogeographic analysis based on grid cells of Cixiidae in China and adjacent areas, including all the available data for the family. However this dataset has is own limits: 1) the stronger collecting efforts into southern China and taxonomic studies clearly advanced in the Taiwan region because of studies by Tsaur over the past three decades (Tsaur and Lee 1987, Tsaur et al. 1988, Tsaur 1989a, Tsaur 1989b, Tsaur 1990a, Tsaur 1990b, Tsaur et al. 1991a, Tsaur et al. 1991b, Tsaur and Hsu 2003, Tsaur 2009), 2) the limited to very limited knowledge of Cixiidae in countries adjacent to China, despite studies by Distant (1911), Emeljanov (1974), Fennah (1978), Anufriev (1987), Anufriev and Emeljanov (1988), Hoch (2013), Anonymous (2015), 3) it does not take into account host-plants, which are however key factors also affecting the distribution of these obligatory, phytophagous planthoppers, although host-plants and the planthopper species complex are also together affected by other complex topographic and climatic factors embedded in a long dynamic geological process. Accordingly, if the high diversity of Chinese Cixiidae - no less than 8.6% of the current total species richness of the family (Bourgoin, 2021) - is probably related to the high diversity of Chinese biotopes, the figures presented here probably over-estimate the level of endimicity of the fauna in comparison with the adjacent countries.

With the current available data, the observed distribution patterns reveals that an intercalary Sino-Japanese realm is recognizable between the Palaearctic and Oriental realms. At the regional level, the South China region clusters more closely with the Southwest, Central and North China regions. Taiwan is clearly separated from the South China region and mainland China, but is more closely related to the Qinghai-Tibet region and Indochina countries. The Central and South China regions are close to each other, but the Qinghai-Tibet region is singularly different. However a much better knowledge of the cixiid fauna in the adjacent countries will be needed in the future for a better evaluation and analysis of the singularity of the Chinese fauna. Additionnaly, a yet to be done phylogenetic analysis of the Cixiidae family will be essential to provide the frame of reference allowing to support any reliable historical biogeography scenario of the evolution, development, and distribution of Cixiidae in China.

## Supplementary Material

A7916101-DDA3-52BA-8A6B-B4CC24C5DA65 10.3897/BDJ.10.e75303.suppl1 Supplementary material 1 48 additional Cixiidae species from adjacent areas based on literature and FLOW (Bourgoin, 2021).
Data type Table Brief description Presence (1) or absence (0) of 48 Cixiidae species in VM (Bangladesh, Bhutan, Cambodia, Laos, Myanmar, Thailand, Vietnam) and RFE (Russian Far East).
* BD, Bangladesh; BT, Bhutan; KH, Cambodia; LA, Laos; MM, Myanmar; TH, Thailand; VN, Vietnam. File: oo_611209.docx https://binary.pensoft.net/file/611209 Yang Luo, Thierry Bourgoin, Ja-Lin Zhang and Ji-Nian Feng

5023400B-A2A4-570E-888A-A3CC3A67E904 10.3897/BDJ.10.e75303.suppl2 Supplementary material 2 The observed material information of checklist Data type spreadsheet Brief description Excel version of the observed specimen information for Checklist. File: oo_619349.xlsx https://binary.pensoft.net/file/619349

## Figures and Tables

**Table 1.** Species richness, endemism and the proportion of endemic species in the 8 zoogeographical regions of China.

**Zoogeographical regions**	**Species richness**	**Number of endemic species**	**Endemic species** %
South China	78	14	17.95
Southwest China	43	17	39.53
Central China	60	20	33.33
North China	29	10	34.48
Northeast China	8	1	12.5
Nei Mongol-Xinjiang	5	0	0
Qinghai-Tibet	10	2	20.00
Taiwan	161	112	69.57
China	250	205	82.00

**Table 2.** Distribution patterns of Cixiidae among China zoogeographical regions and proportion of species in these patterns of the total number of species. * Abbreviations: NEC, Northeast China; NC, North China; NX, Nei Mongol-Xinjiang; QT, Qinghai-Tibet; SWC, Southwest China; CC, Central China; SC, South China; TW, Taiwan.

**Distributed pattern**	**Number of species**	**Species number** %
TW	112	44.80
CC	20	8.00
SWC	17	6.80
SC	14	5.60
NC	10	4.00
QT	2	0.80
NEC	1	0.40
SC-TW	15	6.00
SWC-SC	3	1.20
SWC-CC	3	1.20
CC-SC	2	0.80
NC-CC	2	0.80
CC-TW	1	0.40
NEC-NC	1	0.40
NC-SC	1	0.40
NEC-NX	1	0.40
CC-SC-TW	11	4.40
SWC-SC-TW	6	2.40
NC-SC-CC	2	0.80
SWC-SC-CC	2	0.80
NC-SC-TW	2	0.80
NX-SC-TW	2	0.80
NC-QT-CC	1	0.40
NX-CC-SC	1	0.40
QT-SC-TW	1	0.40
NC-SWC-CC-SC	2	0.80
NEC-CC-SC-TW	2	0.80
NC-CC-SC-TW	1	0.40
QT-SWC-CC-SC	1	0.40
SWC-CC-SC-TW	1	0.40
NC-SWC-CC-SC-TW	2	0.80
NEC-NC-SWC-CC-SC	1	0.40
NC-QT-SWC-CC-SC	1	0.40
NEC-QT-CC-SC-TW	1	0.40
QT-SWC-CC-SC-TW	1	0.40
NEC-NC-SWC-CC-TW	1	0.40
NC-QT-SWC-CC-SC-TW	1	0.40
NC-NX-QT-SWC-CC-SC-TW	1	0.40

**Table 3.** Number and percentage of cixiid species distributed in China by tribes among the Chinese zoogeographical regions. Abbreviations: NEC, Northeast China; NC, North China; NX, Nei Mongol-Xinjiang; QT, Qinghai-Tibet; SWC, Southwest China; CC, Central China; SC, South China; TW, Taiwan.

**Chinese tribes of Cixiidae**	**Number of species**	**Species** %	**Zoogeographical distribution**
Andini Emeljanov, 2002	13	5.20	NC, QT, SWC, CC, SC, TW
Bennini Metcalf, 1938	1	0.40	TW
Brixiini Emeljanov, 2002	2	0.80	TW
Cixiini Spinola, 1839	113	45.20	NC, NX, QT, SWC, CC, SC, TW
Eucarpiini Emeljanov, 2002	16	6.40	SWC, CC, SC, TW
Oecleini Muir, 1922	3	1.20	TW
Pentastirini Emeljanov, 1971	53	21.20	NEC, NC, NX, QT, SWC, CC, SC, TW
Semonini Emeljanov, 2002	43	17.20	NEC, NC, NX, QT, SWC, CC, SC, TW
Stenophlepsiini Metcalf, 1938	1	0.40	TW
Borysthenini Emeljanov, 1989	5	2.00	SWC, CC, SC, TW

**Table 4.** Number and percentage of cixiid genus and species distributed in China by genera amongst the Chinese zoogeographical regions. Abbreviations: NEC, Northeast China; NC, North China; NX, Nei Mongol-Xinjiang; QT, Qinghai-Tibet; SWC, Southwest China; CC, Central China; SC, South China; TW, Taiwan.

**Chinese genera of Cixiidae**	**Number of species**	**Species**	**Zoogeographical distribution**
		%	
*Andes* Stål, 1866	10	4	NC, QT, SWC, CC, SC, TW
*Andixius* Emeljanov & Hayashi, 2007	3	1.2	SWC, TW
*Ankistrus* Tsaur & Hsu, 1991	7	2.8	TW
*Arosinus* Emeljanov, 2007	2	0.8	TW
*Atretus* Emeljanov, 2007	5	2	CC, SC, TW
*Bajauana* Distant, 1907	2	0.8	CC, SC
*Betacixius* Matsumura, 1914	25	10	NC, NX, SWC, CC, SC, TW
*Borysthenes* Stål, 1866	5	2	SWC, CC, SC, TW
*Brixia* Stål, 1859	2	0.8	TW
*Cixius* Latreille, 1804	95	38	NC, NX, QT, CC, SC, TW
*Dilacreon* Fennah, 1980	1	0.4	SC
*Eucarpia* Walker, 1857	4	1.6	SC, TW
*Euryphlepsia* Muir, 1922	1	0.4	TW
*Gonophallus* Tsaur & Hsu, 1991	1	0.4	TW
*Haplaxius* Fowler, 1904	1	0.4	CC
*Indolipa* Emeljanov, 2001	7	2.8	NC, QT, SWC, CC, SC, TW
*Kirbyana* Distant, 1906	4	1.6	SWC, CC, SC, TW
*Kotonisia* Matsumura, 1938	1	0.4	TW
*Kuvera* Distant, 1906	18	7.2	NEC, NC, NX, QT, SWC, CC, SC, TW
*Macrocixius* Matsumura,1914	4	1.6	SWC, CC, SC, TW
*Melanoliarus* Fennah, 1945	2	0.8	CC, TW
*Myndus* Stål, 1862	1	0.4	TW
*Neocarpia* Tsaur & Hsu, 2003	5	2	SWC, CC, TW
*Oecleopsis* Emeljanov, 1971	14	5.6	NC, SWC, CC, SC, TW
*Oliarus* Stål, 1862	6	2.4	NEC, NC, SWC, CC, SC, TW
*Oliparisca* Emeljanov, 2001	1	0.4	QT
*Oteana* Hoch, 2006	1	0.4	TW
*Pentastiridius* Kirschbaum, 1868	5	2	NEC, NC, NX, SWC, CC, SC, TW
*Reptalus* Emeljanov, 1971	6	2.4	NEC, NC, QT, SWC, CC, SC
*Semicixius* Tsaur & Hsu, 1991	1	0.4	TW
*Siniarus* Emeljanov, 2007	3	1.2	NC, QT, SWC, CC, SC, TW
*Tsauria* Kocak & Kemal, 2009	5	2	CC, TW
*Mundopa* Distant, 1906	1	0.4	TW
