# Supplementary material for: Distribution patterns of Chinese Cixiidae (Hemiptera, Fulgoroidea), highlight their high endemic diversity
Source: Biodivers Data J. 2022 Jan 24;10:e75303. doi: 10.3897/BDJ.10.e75303 (PMC8803750; doi:10.3897/BDJ.10.e75303)
Supplement: Supplementary material 1 — 48 additional Cixiidae species from adjacent areas based on literature and FLOW (Bourgoin, 2021). [file bdj-10-e75303-s001.docx]

**Appendix:** Presence (1) or absence (0) of 48 Cixiidae species in VM (Bangladesh, Bhutan, Cambodia, Laos, Myanmar, Thailand, Vietnam) and RFE (Russian Far East).

|  | RFE | VM | VN | LA | TH | KH | MM | BH | BA |
| --- | --- | --- | --- | --- | --- | --- | --- | --- | --- |
| *Plecophlebus nebulosus†* Cockerell, 1917 | 0 | 1 | 0 | 0 | 0 | 0 | 1 | 0 | 0 |
| *Bangoliarus truncatus* Shakila, 1984 | 0 | 1 | 0 | 0 | 0 | 0 | 0 | 0 | 1 |
| *Borysthenes diversa* (Distant, 1906) | 0 | 1 | 0 | 0 | 0 | 0 | 1 | 0 | 0 |
| *Borysthenes nicanor* Fennah, 1978 | 0 | 1 | 1 | 0 | 0 | 0 | 0 | 0 | 0 |
| *Borysthenes strigipennis* Distant, 1911 | 0 | 1 | 0 | 0 | 0 | 0 | 0 | 1 | 0 |
| *Borysthenes suknanicus* Distant, 1911 | 0 | 1 | 0 | 0 | 0 | 0 | 0 | 0 | 1 |
| *Andes hemina* Fennah, 1978 | 0 | 1 | 1 | 0 | 0 | 0 | 0 | 0 | 0 |
| *Andes truncatus* Synave, 1953 | 0 | 1 | 1 | 0 | 0 | 0 | 0 | 0 | 0 |
| *Benna wareea* Hoch, 2013 | 0 | 1 | 0 | 0 | 1 | 0 | 0 | 0 | 0 |
| *Sanghabenna chana* Hoch, 2013 | 0 | 1 | 1 | 0 | 0 | 0 | 0 | 0 | 0 |
| *Sanghabenna dima* Hoch, 2013 | 0 | 1 | 1 | 0 | 0 | 0 | 0 | 0 | 0 |
| *Sanghabenna florenciana* Hoch & Bourgoin, 2017 | 0 | 1 | 1 | 0 | 0 | 0 | 0 | 0 | 0 |
| *Sanghabenna thaya* Hoch, 2013 | 0 | 1 | 1 | 0 | 0 | 0 | 0 | 0 | 0 |
| *Melandeva drymothea* Emeljanov, 2007 | 0 | 1 | 1 | 0 | 0 | 0 | 0 | 0 | 0 |
| *Anila fuliginosa* Distant, 1906 | 0 | 1 | 0 | 0 | 0 | 0 | 1 | 0 | 0 |
| *Cixius (Ussuricixius) remmi* Vilbaste, 1969 | 1 | 0 | 0 | 0 | 0 | 0 | 0 | 0 | 0 |
| *Macrocixius giganteus* Matsumura,1914 | 0 | 1 | 1 | 0 | 0 | 0 | 0 | 0 | 0 |
| *Macrocixius gigantomimus* Orosz, 2013 | 0 | 1 | 1 | 0 | 0 | 0 | 0 | 0 | 0 |
| *Macrocixius grossus* Tsaur & Hsu, 1991 | 0 | 1 | 1 | 0 | 0 | 0 | 0 | 0 | 0 |
| *Macrocixius monticola* Orosz, 2013 | 0 | 1 | 1 | 0 | 0 | 0 | 0 | 0 | 0 |
| *Kirbyana pratti thyas* Fennah, 1978 | 0 | 1 | 1 | 0 | 0 | 0 | 0 | 0 | 0 |
| *Pterolophus anichkini* Emeljanov, 2013 | 0 | 1 | 1 | 0 | 0 | 0 | 0 | 0 | 0 |
| *Mnemosyne laticara* Van Stalle, 1988 | 0 | 1 | 1 | 0 | 0 | 0 | 0 | 0 | 0 |
| *Mnemosyne punctipennis* (Distant, 1906) | 0 | 1 | 0 | 0 | 0 | 0 | 1 | 0 | 0 |
| *Mundopa dohertyi* Distant, 1906 | 0 | 1 | 0 | 0 | 0 | 0 | 1 | 0 | 0 |
| *Mundopa fasciata* Distant, 1906 | 0 | 1 | 0 | 0 | 0 | 0 | 1 | 0 | 0 |
| *Mundopa kotoshonis* Matsumura, 1914 | 0 | 1 | 0 | 0 | 0 | 0 | 1 | 0 | 0 |
| *Mundopa myittae* Distant, 1906 | 0 | 1 | 0 | 0 | 0 | 0 | 1 | 0 | 0 |
| *Indolipa binghami* (Distant, 1911) | 0 | 1 | 0 | 0 | 0 | 0 | 1 | 0 | 0 |
| *Indolipa fusconebulosus* (Distant, 1906) | 0 | 1 | 0 | 0 | 0 | 0 | 1 | 0 | 0 |
| *Oecleopsis artemisiae* (Matsumura, 1914) | 1 | 0 | 0 | 0 | 0 | 0 | 0 | 0 | 0 |
| *Oecleopsis petasatus* (Noualhier, 1896) | 0 | 1 | 0 | 0 | 0 | 1 | 0 | 0 | 0 |

**Appendix:** (Cont.)

|  | RFE | VM | VN | LA | TH | KH | MM | BH | BA |
| --- | --- | --- | --- | --- | --- | --- | --- | --- | --- |
| *Oecleopsis sinicus* (Jacobi, 1944) | 0 | 1 | 0 | 0 | 0 | 1 | 0 | 0 | 0 |
| *Oecleopsis yoshikawai* (Ishihara, 1961) | 0 | 1 | 0 | 0 | 1 | 0 | 0 | 0 | 0 |
| *Oecleus cucullatus* (Noualhier, 1896) | 0 | 1 | 0 | 0 | 0 | 1 | 0 | 0 | 0 |
| *Oliarus annandalei* Distant, 1911 | 0 | 1 | 1 | 0 | 0 | 0 | 0 | 0 | 0 |
| *Oliarus tectonae* Shakila, 1984 | 0 | 1 | 0 | 0 | 0 | 0 | 0 | 0 | 1 |
| *Pentastiridius apicalis* (Uhler, 1896) | 1 | 0 | 0 | 0 | 0 | 0 | 0 | 0 | 0 |
| *Pentastiridius kaszabianus* (Dlabola, 1970) | 1 | 0 | 0 | 0 | 0 | 0 | 0 | 0 | 0 |
| *Pentastiridius leporinus* (Linné, 1761) | 1 | 0 | 0 | 0 | 0 | 0 | 0 | 0 | 0 |
| *Betacixius pallidior* Jacobi, 1944 | 0 | 1 | 1 | 0 | 0 | 0 | 0 | 0 | 0 |
| *Betacixius tonkinensis* Matsumura, 1914 | 0 | 1 | 1 | 1 | 0 | 1 | 0 | 0 | 0 |
| *Kuvera flaviceps* Matsumura, 1914 | 1 | 0 | 0 | 0 | 0 | 0 | 0 | 0 | 0 |
| *Kuvera kurilensis* Anufriev, 1987 | 1 | 0 | 0 | 0 | 0 | 0 | 0 | 0 | 0 |
| *Kuvera pallidula* Matsumura, 1914 | 1 | 0 | 0 | 0 | 0 | 0 | 0 | 0 | 0 |
| *Kuvera semihyalina* Distant, 1906 | 0 | 1 | 0 | 0 | 0 | 0 | 1 | 0 | 0 |
| *Kuvera ussuriensis* (Vilbaste, 1968) | 1 | 0 | 0 | 0 | 0 | 0 | 0 | 0 | 0 |
| *Kuvera vilbastei* Anufriev, 1987 | 1 | 0 | 0 | 0 | 0 | 0 | 0 | 0 | 0 |

* BD, Bangladesh; BT, Bhutan; KH, Cambodia; LA, Laos; MM, Myanmar; TH, Thailand; VN, Vietnam.
